# Whole Exome Sequencing of Patients from Multicase Families with Systemic Lupus Erythematosus Identifies Multiple Rare Variants

**DOI:** 10.1038/s41598-018-26274-y

**Published:** 2018-06-08

**Authors:** Angélica M. Delgado-Vega, Manuel Martínez-Bueno, Nina Y. Oparina, David López Herráez, Helga Kristjansdottir, Kristján Steinsson, Sergey V. Kozyrev, Marta E. Alarcón-Riquelme

**Affiliations:** 1 Department of Immunology, Genetics and Pathology, Uppsala University, The Rudbeck Laboratory, Uppsala, Sweden; 2 Pfizer/University of Granada/Andalusian Government Centre for Genomics and Oncological Research (GENYO), Granada, Spain; 3 Institute for Environmental Medicine, Karolinska Institutet, Solna, Sweden; 4 Science for Life Laboratory, Department of Medical Biochemistry and Microbiology, Uppsala University, Uppsala, Sweden; 5 Department Effect-Directed Analysis, Helmholtz Centre for Environmental Research - UFZ, Leipzig, Germany; 6 Unit of Rheumatology, Landspitalinn, Reykjavik, Iceland

## Abstract

In an effort to identify rare alleles associated with SLE, we have performed whole exome sequencing of the most distantly related affected individuals from two large Icelandic multicase SLE families followed by Ta targeted genotyping of additional relatives. We identified multiple rare likely pathogenic variants in nineteen genes co-segregating with the disease through multiple generations. Gene co-expression and protein-protein interaction analysis identified a network of highly connected genes comprising several loci previously implicated in autoimmune diseases. These genes were significantly enriched for immune system development, lymphocyte activation, DNA repair, and V(D)J gene recombination GO-categories. Furthermore, we found evidence of aggregate association and enrichment of rare variants at the *FAM71E1/EMC10* locus in an independent set of 4,254 European SLE-cases and 4,349 controls. Our study presents evidence supporting that multiple rare likely pathogenic variants, in newly identified genes involved in known disease pathogenic pathways, segregate with SLE at the familial and population level.

## Introduction

Systemic lupus erythematosus (SLE [MIM:152700]) is a chronic and systemic autoimmune disease that affects primarily women (90%) during their reproductive years^1^. Clinically, SLE has heterogeneous manifestations ranging from skin rash and arthritis, through anemia and thrombocytopenia, to serositis, nephritis, seizures, and even psychosis^2,3^. The hallmark of SLE is the production of autoantibodies by autoreactive B-cells against multiple cellular components, especially nucleic acids and their interacting proteins. The deposition of immune complexes and widespread inflammation lead to multiple organ damage. Although the pathogenic mechanisms leading to the breakdown of immune tolerance in SLE are not completely understood, it is well established that it depends on multiple genetic, epigenetic, hormonal, and environmental factors; therefore, it is a complex disease^2,3^.

While autoimmune diseases together affect around 3–5% of the world population^4^, SLE is relatively uncommon. Its prevalence varies widely between populations, reflecting the effect of population-specific genetic and environmental factors (i.e. diet, UV radiation). In 1984 the prevalence of SLE in the Icelandic population was estimated to be 35.9 per 100,000 individuals with an overall incidence of 3.3 cases per 100,000 per year, similar to other North European populations^1,5^. In contrast, the risk of developing SLE among siblings of patients is up to ~30 times the risk of the general population (λs = 8–29)^6^, and approximately 8–12% of all SLE cases have a first, second or third degree relative with the disease^6^.

Early family-based studies of SLE revealed high heritability (~66%) and a most probable model of inheritance in which multiple minor polygenic effects were acting in an additive fashion^7,8^. Genetic linkage studies of multicase families provided the first evidence about the location of susceptibility genes for SLE^9–14^. Over the last 10 years, an explosion of large case-control genome-wide association studies (GWAS) have provided strong evidence of association for common variants (minor allelic frequency (MAF) >1%) in over 50 loci^15–25^ making clear that SLE is a polygenic disease, although rare Mendelian forms of SLE-like disorders have been described^26–29^. The identification of these genes has greatly contributed to the understanding of the disease pathogenesis establishing that innate and adaptive immune genes are primarily involved^25^. However, the variants identified so far by GWAS explain about 10–19% of the heritability^16,25,30^. As GWAS has focused on common variants, we still do not know the relative impact of rare variants, or their role in the development of sporadic and familial SLE.

**Figure 1:**
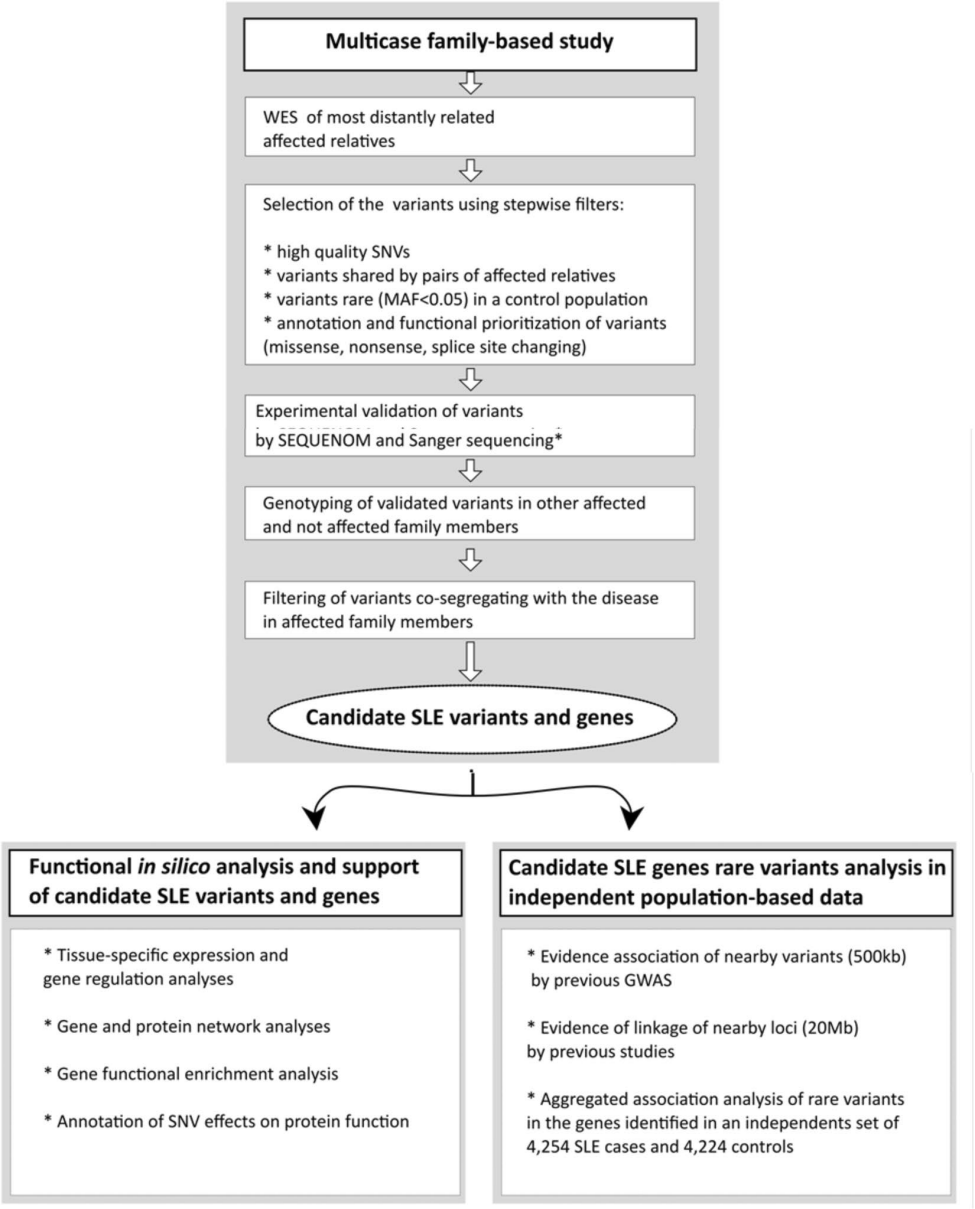
Graphic summary of the study design. Step-by-step scheme shows initial variant detection, validation and filtering followed by *in silico* functional analysis of candidate loci and analysis of candidate genes in independent population-based data.

Thanks to the rapid development of next generation DNA sequencing (NGS) technologies, it is now feasible and affordable to use whole exome sequencing (WES) or even whole genome sequencing (WGS) to systematically interrogate virtually all coding variants in the human genome. Thus, in an effort to study the role of rare variation in SLE, we analysed WES data from five patients from two large well-studied Icelandic SLE multi-case families, for which we have clinical and linkage data. We interrogated whether rare, likely pathogenic variants were co-segregating with the disease through multiple generations by sequencing the most distantly related individuals in each family and then performed a genotyped-based follow-up of the variants identified in other affected family members. Interestingly, we did not find single alleles in the multi-case families, but instead we found groups of rare alleles in each family segregating with disease. These genes were further investigated by imputation of sporadic SLE GWAS data, and various omic strategies were implemented to identify and predict pathogenic networks comprised by these genes. After applying very stringent criteria to correct for the potential effect of population stratification and linkage disequilibrium, we found evidence of enrichment and aggregate association for a new locus in an independent set of 4,254 European SLE cases and 4,349 controls. The set of genes was significantly enriched for biological processes such as immune system development, lymphocyte activation, and DNA metabolic processes including DNA repair and V(D)J gene recombination. A graphic representation of the study design is presented on Fig. 1.

## Results

### Exome Sequencing and Variant Filtering

We successfully sequenced the exome of the most distantly related patients with SLE (n = 5) from two well-studied multi-case SLE families from Iceland (Fig. 1 and Table S1)^31^ to a mean read depth of 33.3X across targeted coding regions (Table S2). We chose a filtering strategy favouring the best-quality single nucleotide variants (SNVs) shared by all the affected sequenced individuals from each family, with a very low allele frequency (MAF < 0.01) in an internal control population (n = 642), and with easily recognized functional consequences for protein coding genes (gain or loss of a stop codon, nonsense, missense, and essential splice sites). A total of nineteen variants passed all quality-, annotation- and frequency-based filtering criteria, and were validated by SEQUENOM and/or Sanger sequencing (Table 1). All the 19 identified variants were non-synonymous SNVs (nsSNVs) present in heterozygous state in the exome-sequenced patients. No variant with a gene truncating effect (stop gain, stop loss, nonsense, or essential splice variant) was identified.

**Table 1:** Segregation analysis and population frequency for exome-sequence variants.

Variant number	Gene	Chromosome position, hg19	Protein change	rsid	Top functional categories (pathways//GO biological process)	Expression in GTEX blood/immune samples	Family 6 carriers/cases	Family 8 carriers/cases	Linkage within 20 Mb Family 6 (Z-score)	Linkage within 20 Mb Iceland (Z-score)	Controls	Nordic controls	Maximum MAF in public databases (global population)
1	*ANKRD50*	chr4:125593332	T367M	rs140232140	Transport; protein transport; retrograde transport, endosome to plasma membrane	**Yes**	3/8	—	—	—	0.0024	—	0.0091
2	*CHD3*	chr17:7810250	A1523T	rs148451716	Activated PKN1 stimulates transcription of AR (androgen receptor) regulated genes KLK2 and KLK3; Chromatin organization; Gene expression//chromatin organization; chromatin assembly or disassembly; transcription, DNA-templated	**Yes**	4/8	—	—	1.79	0	—	0.0003
3	*FAT4*	chr4:126238305	P247T	rs191329848	Hippo signaling pathway//branching involved in ureteric bud morphogenesis; kidney development; heart morphogenesis; plasma membrane organization	No	3/8	—	—	—	0.0016	0.0061	0.0042
4	*KIR2DS4*	chr19:55358686	I255L	rs112697729	**Innate immune system; Allograft rejection; Immune response Role of DAP12 receptors in NK cells**//**innate immune response**	**Yes**	6/8	1/7	—	2.06	0	0.1689	—
5	*NUP214*	chr9:134027138	I765V	rs61756081	**HIV Life Cycle; Cell Cycle, Mitotic; Mitotic Prophase; Influenza Viral RNA Transcription and Replication**//**regulation of glycolytic process; RNA export from nucleus**	**Yes**	3/8	—	1.3	—	—	0.012	0.0097
6	*PDHA2*	chr4:96762158	R286P	rs147966234	Citrate cycle (TCA cycle); Glucose metabolism; Carbon metabolism//carbohydrate metabolic process; glucose metabolic process	No	3/8	—	—	—	0.0071	0.0241	0.0091
7	*SCL25A9*	chr1:48694594	G103R	rs61746559	Transport of glucose and other sugars, bile salts and organic acids, metal ions and amine compounds; Hexose transport//transport; ion transport	No	4/8	2/7	1.13	2.4	0.0071	0.006	0.0525
8	*XRCC6BP1*	chr12:58350618	A229V	rs117230607	Double-strand break repair via nonhomologous end joining; protein phosphorylation; proteolysis	**Yes**	6/8	—	—	—	0.0063	0.0183	0.0053
9	*TPRA1*	chr3:127292588	E300K	rs372625321	Lipid metabolic process; G-protein coupled receptor signaling pathway; aging; negative regulation of mitotic cell cycle phase transition	**Yes**	—	3/7	—	—	—	—	0.00008
10	*KRTAP4-9*	chr17:39261693	D18V	rs113059833	Aging; keratinization; hair cycle	No	8/8	4/7	—	—	0	0.3765	0.1879
11	*MPHOSPH8*	chr13:20224319	E499K	rs147594834	Transcription, DNA-templated; regulation of transcription, DNA-templated; regulation of DNA methylation; negative regulation of transcription, DNA-templated	**Yes**	—	3/7	—	—	0.008	—	0.003
12	*NOTCH1*	chr9:139404360	D932N	rs758642073	**Signaling by NOTCH1; HIV life cycle**//**negative regulation of transcription from RNA polymerase II promoter; angiogenesis; in utero embryonic development; cell fate specification**	**Yes**	—	5/7	—	—	0	—	0.0002
13	*PABPC3*	chr13:25670676	A114T	rs117014540	mRNA surveillance pathway; Deadenylation-dependent mRNA decay; RNA transport//mRNA metabolic process	No	—	3/7	—	—	0.0094	0.012	0.0044
14	*WDR25*	chr14:100847878	R206H	rs146976933	_//_	**Yes**	—	3/7	—	—	0.0031	—	0.008
15	*CLC*	chr19:40225031	N65K	rs146776010	**Regulation of T cell anergy; regulation of T cell cytokine production; regulation of activated T cell proliferation**	**Yes**	—	5/7	—	2.06	0.0071	0.006	0.0176
16	*DCLRE1C*	chr10:14970085	H283N	rs772438042	**DNA Double-Strand Break Repair; Primary immunodeficiency; DNA Damage**//**telomere maintenance; adaptive immune response; immune system process; DNA repair; double-strand break**	**Yes**	—	5/7	1	—	0	—	0.000008
17	*FAM71E1*	chr19:50978724	L7F	rs185418641	_//_	No	—	4/7	—	2.06	0.0063	0.0061	0.0073
18	*FBXL14*	chr12:1702929	N102H	rs117331652	**Class I MHC mediated antigen processing and presentation; Innate Immune System**//**protein polyubiquitination; protein ubiquitination involved in ubiquitin-dependent protein catabolic process; post-translational protein modification**	**Yes**	—	5/7	—	—	0.0055	—	0.0049
19	*FAM8A1*	chr6:17601340	G234R	rs202036280	_//_	**Yes**	—	3/7	—	—	—	—	0.00005

Table 1 shows the results from the segregation analysis for the variants identified by WES as the number of family members who were variant carriers affected with SLE (family 8) or any autoimmune disease (family 6), over the total number of cases in each family. Z-score is indicated for the variants located in regions linked to SLE in Iceland according to ref.^27^. MAF (minor allelic frequency) of the variants in 642 internal whole-genome sequenced European and 83 Nordic controls. The maximum MAF corresponds to the highest frequency of allele in ExAc, 1000 Genomes and GO-ESP data for global population. Variant numbers correspond to those seen in Fig. 3. Functional annotation is shown according to GeneCards SuperPathways and Gene Ontology top biological process categories. The presence of the particular gene transcripts in GTEX blood/immune samples is marked according to Fig. 3. Additional annotation of the variants shown in Table S3.

### Segregation Analysis

We then performed a segregation analysis by looking at the variants co-segregating with the disease status in other affected members within each family for whom DNA was available. This analysis included five cases with SLE, one case with rheumatoid arthritis (RA) and one with multiple sclerosis (MS) in “family 6” (n = 8), as well as seven cases with SLE in “family 8” (Fig. S1).

All of the 19 identified variants were present in at least three affected members of each family including the exome-sequenced patients. Variants in *KRTAP4-9* (p.D18V), *KIR2DS4* (MIM 604955) (p.I255L), and *SLC5A9* (p.G103R) were the most frequent among patients of both families (12, 7 and 6 patients, respectively). However, the former two variants were very frequent (MAF > 5%) among a set of 83 controls from Iceland and Sweden (referred to as *Nordic controls*) (Tables 1 and S3). Due to the high frequency in the Nordic controls and discrepancies across public databases, these variants were excluded from further detailed analysis.

**Figure 2:**
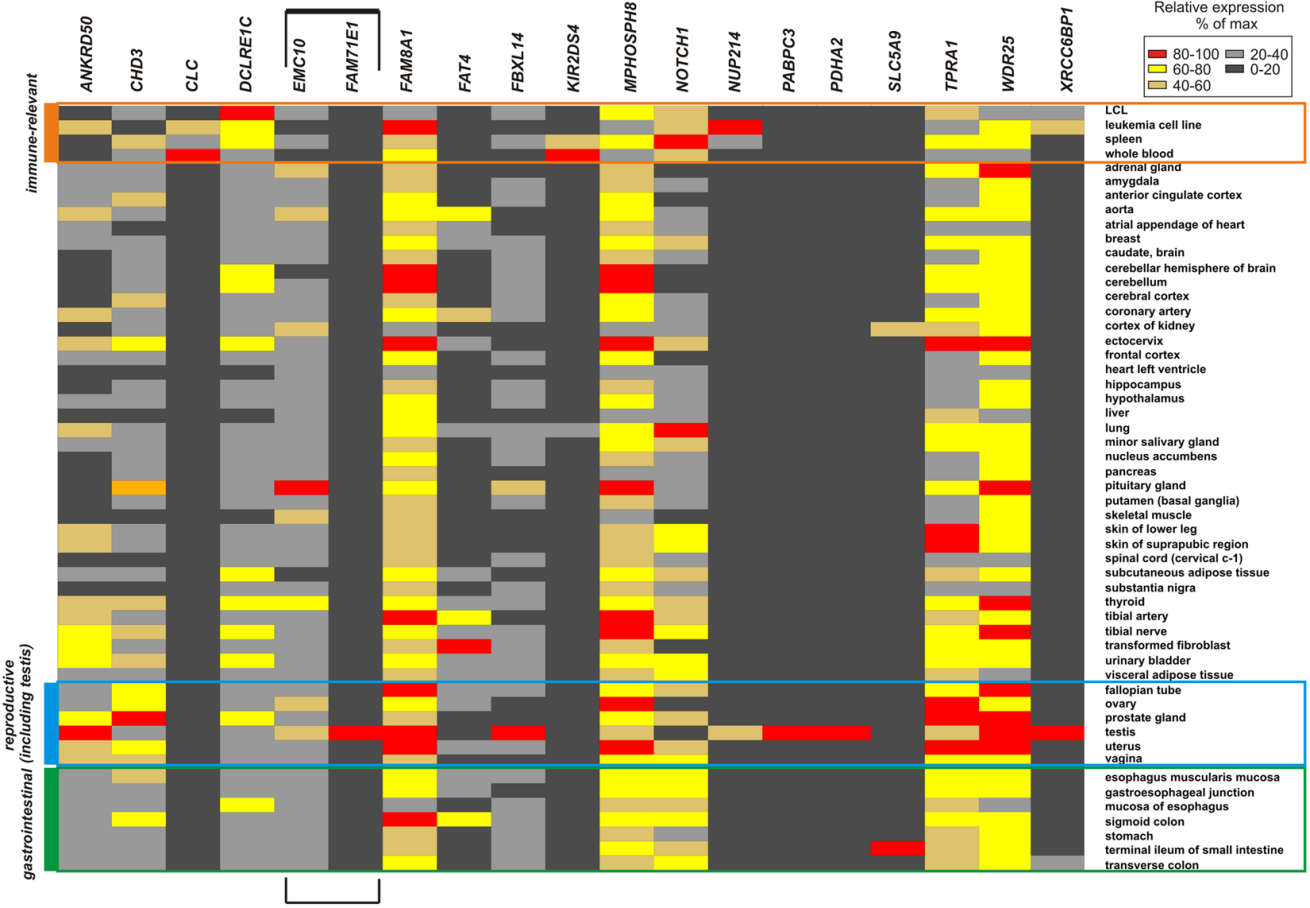
Tissue-specific expression patterns for genes with nsSNVs. The heat plot shows GTEX RNAseq expression levels based on FPKM values. Immune-relevant samples, reproductive system and gastrointestinal tract are outlined in orange, blue and green boxes. For the bidirectional *FAM71E1-EMC10* locus expression pattern of both genes is shown.

We observed the best co-segregation with disease status in family 6 for a variant in *XRCC6BP1* (also known as *ATP23*) (p.A229V), which was carried by six out of eight affected members including four SLE patients, one RA and one MS patient. In other words, the *XRCC6BP1* variant segregated with all autoimmune diseases present in this family. The variants in *SLC5A9* (p.G103R) and *CHD3* (MIM 602120) (p.A1523T) were also carried by four SLE patients; the latter also by one RA patient. In family 8, five out of seven SLE relatives carried the variants in *DCLRE1C* (MIM 605988) (p.H283N), *NOTCH1* (MIM 190198) (p.D932N), *FBXL14* (MIM 609081) (p.N102H) and *CLC* (MIM 153310) (p.N65K) (Table 1). Hence, there was no single variant carried by all SLE patients within each family, but rather a combination of a few rare and low frequency non-synonymous variants segregating with the disease status in most of the affected members. A large fraction of these loci was already annotated as immune-relevant, taking into account known pathways and Gene Ontology: *NOTCH1, KIR2DS4, NUP214, CLC, DCLRE1C* and *FBXL14*. Moreover, 13 of 19 variants-corresponding genes are expressed in whole blood, spleen or relevant cell lines (GTEX RNAseq data, Table 1 and Fig. 2).

### Aggregate Association and Enrichment Analysis

We then tested whether any of the candidate genes identified by exome sequencing had statistical evidence of association with SLE in the general European population due to the combined effect of all rare variation within each gene (MAF < 1%) (Table 2). For this, we used a large and independent imputed genome-wide association scan of 4,254 SLE patients and 4,349 controls with European ancestry^25^ (Table 2). Each gene was analyzed using two procedures: the sequence kernel association test (SKAT)^32^ and an aggregated case-control enrichment test. It is important to note that we performed targeted gene-based tests, that is, we did not test for association of rare variants neither at a genome-wide level nor tested individual variants. To be as stringent as possible, the 10 first principal components (PC’s), accounting for all the significant variability due to population stratification, and genomic control (GC) were used to correct for stratification in both procedures (Figs S2 and S3). To further eliminate the potential effect of linkage disequilibrium (LD) on the computation of empirical corrected P values, tests were run using only unlinked markers by applying a very stringent LD threshold of r² < 0.1. It could be objected that such stringent filters could mask true association signals, but our rationale was that if the signals were maintained after strict correction they would strongly support a ‘true positive’ effect.

**Table 2:** Rare-variant Association Analysis.

***GENE***	*NMK*	Enrichment test		SKAT	
		*Variants with r²* <*0.1, MAF* <*1%* - *Adjusted by 10 PCs and λ*_*GC*_			
		*P*	*Pmult*	*P*	*Pmult*
***DCLRE1C***	58	**3.31E-02**	6.51E-02	5.50E-01	6.54E-01
***EMC10***	41	**1.06E-02**	**2.47E-02**	**2.14E-02**	**3.67E-02**
***FAM71E1***	19	**5.23E-03**	**2.97E-02**	**3.13E-02**	**4.63E-02**

Gene-based case-control association analysis of unlinked rare variants (MAF < 1% and r² < 0.1) of the genes identified by exome sequencing in an independent imputed genome-wide association scan from a set of 4,254 SLE patients and 4,349 controls with European ancestry^25^. Two procedures were used, an enrichment case-control association test and the sequence kernel association test (SKAT). Correction for multiple testing was run through a bootstrapping permutation process (*Pmult*). All tests were corrected for stratification by adjusting for the first 10 principal components (PC) and Genomic Inflation Control (*λ*_*GC*_).

The exome-identified gene *FAM71E1* and the adjacent *EMC10* gene (MIM 614545), which is in high LD, showed significant association with both procedures, case-control enrichment and SKAT, after application of r² < 0.1 threshold and having applied the appropriate corrections for stratification and multiple testing (Table 2). The *DCLRE1C* gene showed suggestive evidence of enrichment but did not remain significant after correction for multiple testing.

### Functional Annotation of Variants and Genes

#### Predicted effect of Non-synonymous SNVs on Protein Function

**Figure 3:**
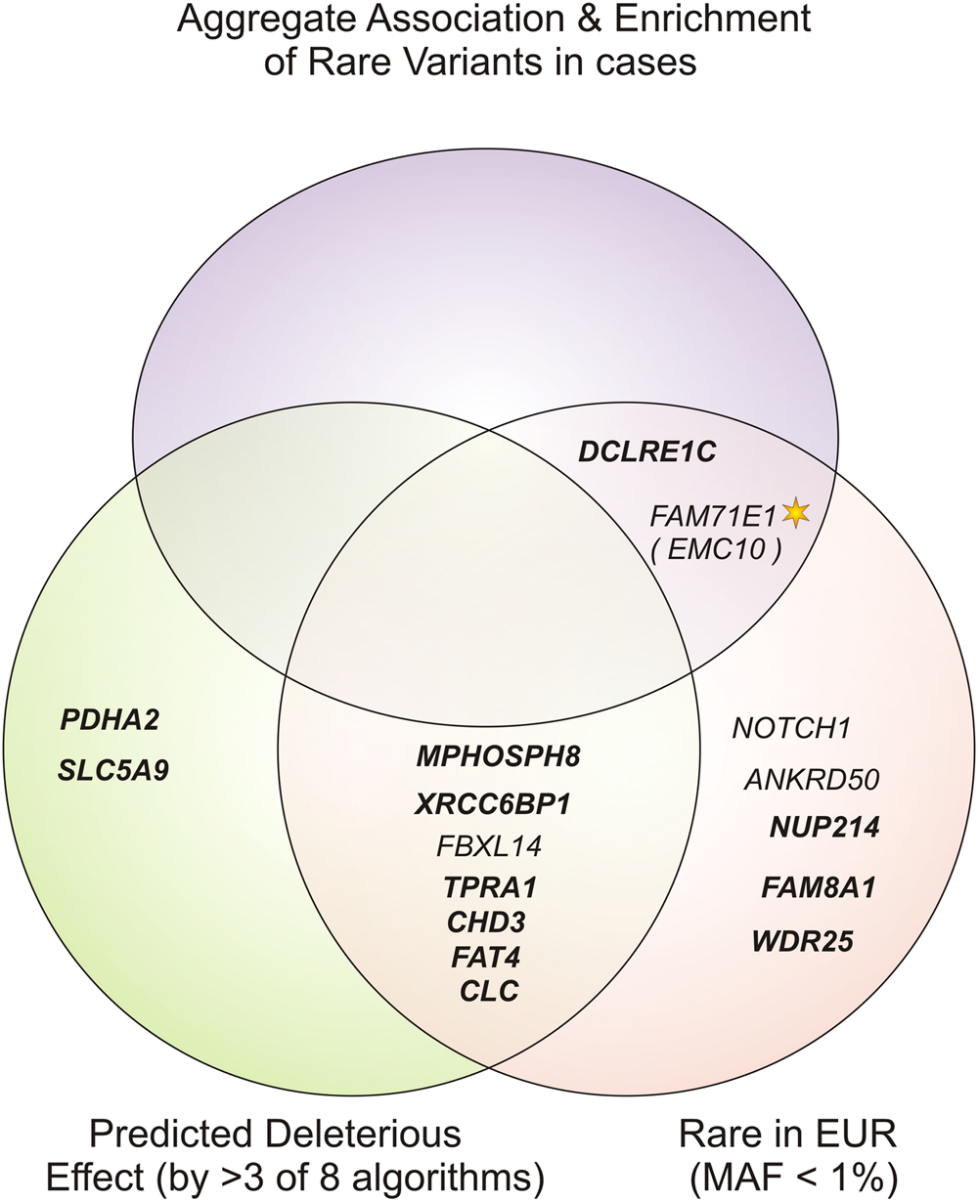
Graphic summary of annotation-based filtering for the variants identified by whole exome sequencing of patients with SLE from multicase families. The Venn diagram shows the genes harbouring non-synonymous variants (nsSNV) with a likely deleterious effect on protein function based on: (Left) a predicted deleterious effect by ≥3 with standard algorithms (SIFT, PolyPhen2, FATHMM, LRT, MetaLR, MutationAccessor, MutationTester, Provean) - genes for which nsSNVs were also predicted as deleterious by one-to-one orthologue-specific SNPDryad algorithm (score > 0.5) are shown in bold - (See detailed scores in Table S3); and (right) a maximum minor allelic frequency of 1% in European populations (internal sequencing and genotyping controls, 1000 Genomes EUR, ESP6500 EurAm and ExAc Eur non-Finish populations) (See Tables S3 and 1). Genes with a significant aggregate association and enrichment of rare variants (top) have further genetic evidence of being implicated in SLE. Genes associated after removing linked variants (r^2^ < 0.1), adjustment by 10 principal components and genomic control, and multiple test correction are highlighted with a star (See Table 2).

The SNPDryad method^33^ and ENSEMBL VEP (Variant Effect Predictor based on SIFT, PolyPhen2, FATHMM, LRT, MetaLR, MutationAccessor, MutationTester, and Provean)^34^, were used for the annotation of the potential deleterious effects of the exome variants. Variants displayed consistent results across all the scoring algorithms (Table S3). Out of the 19 nsSNVs, nine (47%) at *SLC5A9*, *XRCC6BP1*, *MPHOSPH8*, *CHD3*, *CLC*, *TPRA1*, *FAT4*, *PDHA2*, and *FBXL14* were predicted as having a likely deleterious effect by three or more ENSEMBL VEP algorithms, and thirteen nsSNVs (63%) had SNPDryad scores over 0.5 (possibly deleterious: *SLC5A9, DCLRE1C, NUP214, XRCC6BP1, MPHOSPH8, WDR25, CHD3, CLC, TPRA1, ANKRD50, FAT4, PDHA2*, and *FAM8A1*) (Fig. 3 and Table S3). In addition, four genes (*DCLRE1C, NOTCH1, NUP214*, and *FAT4*) were connected to specific phenotypes with immunological features through OMIM (Online Mendelian Inheritance in Man) and the Human Genome Mutation Database (HGMD)^35^. None of the nineteen variants had been reported to ClinVar^36^ (https://www.ncbi.nlm.nih.gov/clinvar/) or HGMD (http://www.hgmd.org) databases (as to Jun 29th, 2017).

Variants in *DCLRE1C* (p.H283N) and *NUP214* (p.I765V) were classified as likely benign by standard algorithms, whereas the SNPDryad method predicted a likely deleterious effect. In addition, these two variants are very rare (<0.5%) in European populations (ESP6500, ExAC and 1000 genomes) and in our internal sequencing controls, which we considered as supporting evidence of a likely gene-disrupting effect (Tables 1 and S3). In contrast, the *SLC5A9* nsSNV (p.G103R) (rs61746559) segregated with SLE in both families, was not common in our dataset and Nordic controls and was predicted to be damaging by several of the algorithms (Table S3), but it shows very high allele frequency in non-European populations (20% in Asians, 26% in Southern Han Chinese −1000 Genomes), which is against a gene-disrupting effect. Likewise, the variants in *KRTAP4-9* (p.D18V) and *KIR2DS4* (p.I255L) were rare (MAF ≤ 1%) in most European populations, but very common in the set of 85 genotyped Nordic controls (≥5%). These common nsSNVs could be polymorphisms specific of the Asian and Nordic population, respectively. We cannot exclude that any of these are functional and/or disease variants, or that the high MAF is due to a founder effect in specific populations, but due to the high frequency and described discrepancies they were regarded as polymorphisms, unlikely to be gene-disrupting variants (Table 1, Fig. 3, and Table S3).

#### Gene Expression Analysis

We interrogated the potential biological relevance of the genes carrying the identified exome variants by analysing their pattern of gene expression, regulation and gene networks. Since the association of *FAM71E1* extends to the nearby gene *EMC10* (Table 2), and they are overlapping and transcribed in opposite directions, we included both genes in the analysis. A heat-map representing the tissue-specific RNAseq data (GTEx Project) for all genes is shown on Fig. 2 and summarized in Table S4. Most of the genes (13 out 19, or 14 out 19, taking into account expression of *FAM71E1-*neighbouring *EMC10*) were expressed in immune-relevant samples. Additionally, several genes are differentially expressed in infections and after specific pathogen exposure (Table S5). We observed no evidence of expression of *KRTAP4-9*, coding for hair keratin-associated protein 4–9, in any of the tissue samples analysed. The expression of *PDHA2*, *PABPC3*, and *FAM71E1* was detected only in testis*. EMC10* was expressed in spleen and lymphoblastoid cell lines, albeit weakly. *FAT4* cadherin as well as the intestinal-specific *SLC5A9* gene, although expressed in several tissues, were almost not detectable in any of the immune-relevant tissues (Fig. 2). In summary, among the exome identified genes the fraction of loci showing immune-relevant expression was high and included the *EMC10* gene from the bidirectional *FAM71E1/EMC10* locus. The tissue-specific expression patterns were independently corroborated using TSS activity data from the FANTOM5 project (Fig. S4).

#### Gene Co-expression and PPI networks

**Figure 4:**
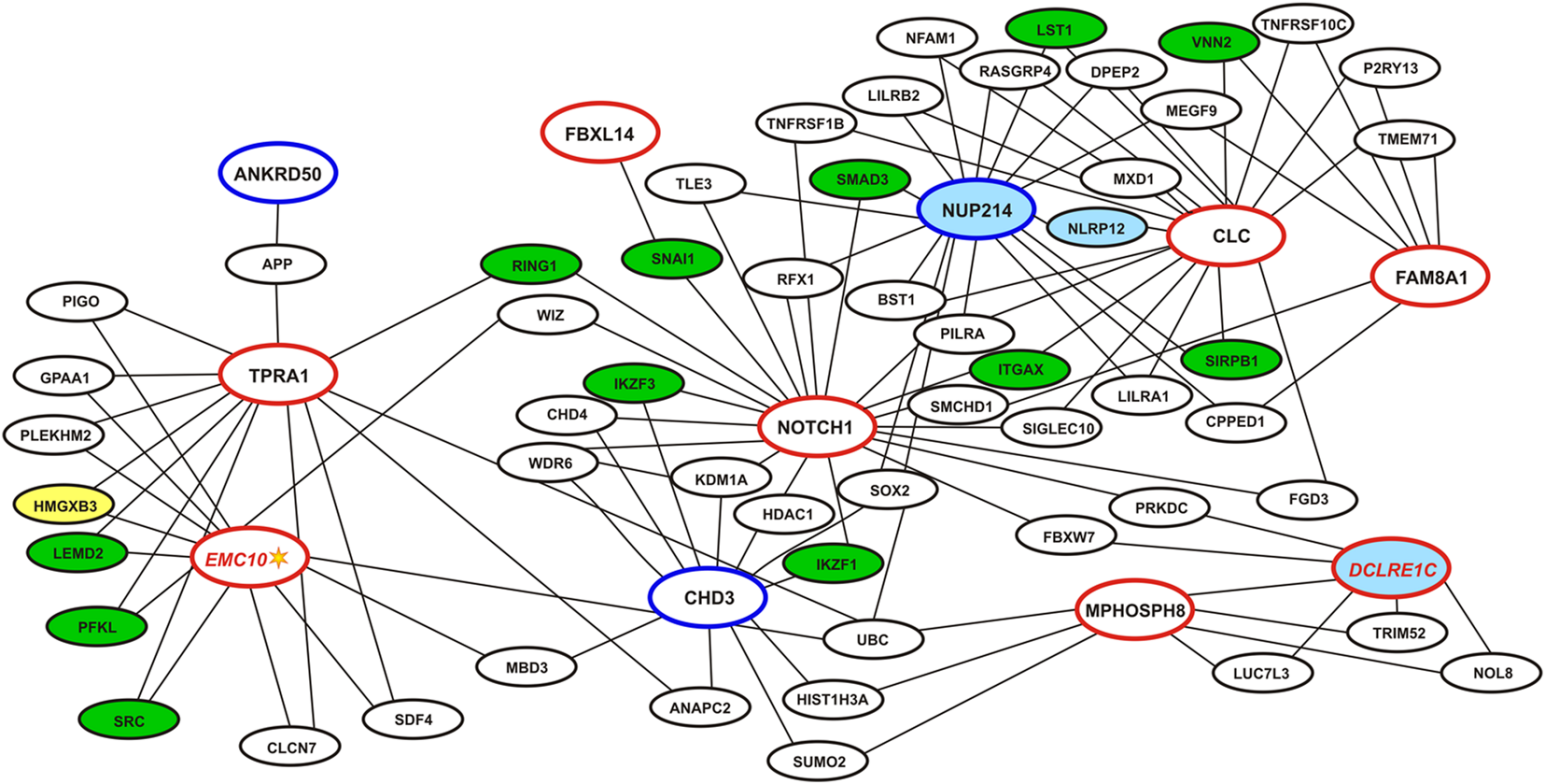
Global pairwise gene interaction network of the exome identified genes. Gene co-expression and direct protein-protein interactions are shown as a combined network. Family 8-specific genes are shown in red ovals, family 6 - in blue ovals. The names of the genes with significant enrichment or aggregated association of rare variants in SLE cases are shown in red italic. Genes associated after removing linked variants (r^2^ < 0.1), adjustment by 10 principal components and genomic control, and multiple test correction are highlighted with a star. Note the high connectivity within the network of the SLE-associated genes. Blue-filled ovals correspond to the genes with known immunity-related Mendelian disorders (OMIM data), green-filled - to the genes with published genome-wide significant associations with autoimmune and autoinflammatory disorders, yellow-filled - with other genome-wide significant immunity-related traits (Table S4).

**Figure 5:**
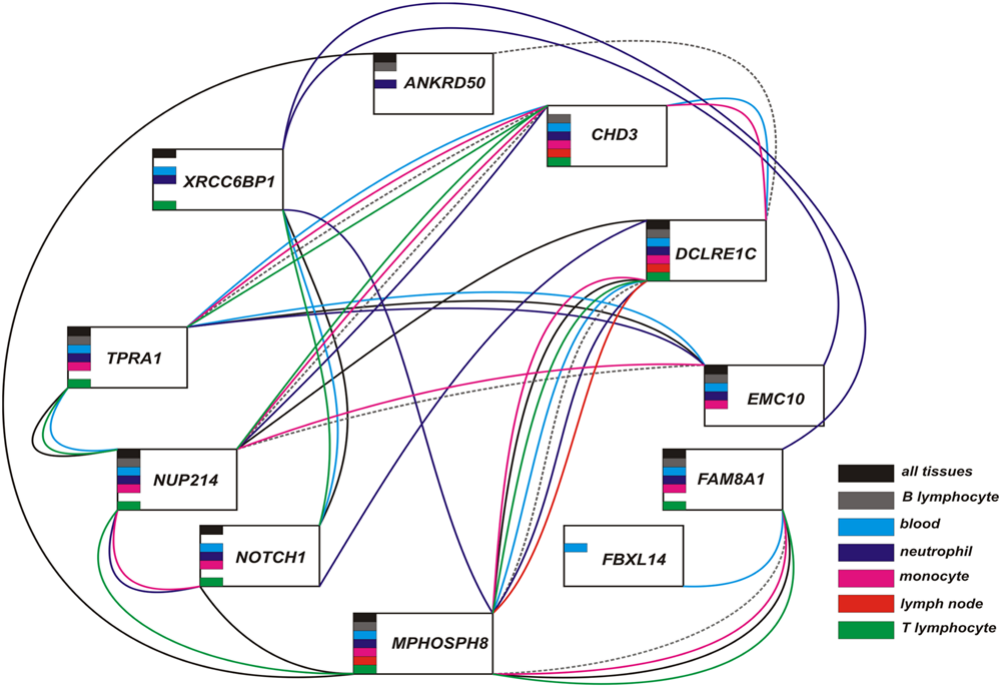
GIANT network for tissue-specific gene connectivity. The connections are shown for “all tissues”, lymph node, blood and for separate blood cell populations and correspond to one or more common partner genes between each of the studied genes. The connecting lines coloured according to the specificity.

We next constructed gene networks based on the detection of common partners between the exome identified genes including *EMC10*. Both gene co-expression and pairwise protein-protein interactions (PPI) were taken into consideration in the networks. We first performed a family-specific analysis. The genes segregating in family 8 were part of a highly connected gene network (Fig. S5). The family-6 gene set was small; nevertheless, the genes *CHD3*, *NUP214* and *FAM8A1* were connected via at least one partner each (Fig. 5). Further on, the gene sets of both families were analysed together (Fig. 4). The resulting network included twelve genes, of which the SLE associated genes *EMC10* and *DCLRE1C*, together with *NUP214*, *CHD3, NOTCH1, FAM8A1*, *MPHOSPH8*, *TPRA1* and *CLC* showed high connectivity to each other and to other genes in the network via several partners. One interaction partner connected genes *ANKRD50* and *FBXL14* each, separately, to the network. Moreover, we observed strong inter-family gene connections: the family 6-specific genes, *CHD3* and *NUP214*, were highly connected to others, including family 8-specific *NOTCH1*, *CLC*, *EMC10*, *TPRA1*, and *MPHOSPH8*.

We independently validated the gene-gene relations by using the tissue-specific GIANT resource based on the unsupervised analysis of public RNAseq data^37^. Of note, *XRCC6BP1*, which was absent in the co-expression and PPI network, was connected to *NOTCH1*, whereas the *CLC* connections were not stable in the GIANT networks (Figs 5 and S6). The difference between results could probably be due to the different calculation algorithms and a contribution of microarray-RNAseq differences in co-expression analysis. However, most of the highly connected genes coincide in the two independent approaches.

Of interest, the networks contain several interaction partners previously genetically implicated in SLE, autoimmune disorders, and other immune-related disorders and traits (Table 3 and Table S6). Importantly, *EMC10* has been consistently associated to the autoimmune disease primary biliary cirrhosis^38–40^.

**Table 3:** Immune-related disorders and traits associated with genes identified through exome sequencing and their partners.

Trait/disorder name	Exome sequencing genes	Potential partner genes
Acute lymphoblastic leukemia	NUP214*(MIM 613065, S)	2 (BCR * [MIM 613065, S]*; IKZF1)*
Acute myeloid leukemia	NUP214*(MIM 601626, S)	
Alloimmunization response to red blood cell transfusion in sickle cell anemia		*1 (ARAP1)*
Antibody status in Tripanosoma cruzi seropositivity		*1 (FARSA)*
Asthma		*3 (CRBN, IKZF3, SMAD3)*
Chronic lymphocytic leukemia		*1 (PRKD2)*
Chronic myeloid leukemia		1 (BCR * [MIM 608232, S]*)*
Clozapine-induced agranulocytosis		*1 (FARSA)*
Crohn’s disease	*NOTCH1*	*8 (IKZF1, IKZF3, LEMD2, LST1, SCAMP3, SMAD3, USP34, VNN2)*
Familial cold autoinflammatory syndrome 2		1 (NLRP12 * [MIM 611762, AD]*)*
Hennekam lymphangiectasia-lymphedema syndrome 2	FAT4* (MIM 616006, AR)	
HIV-1 control		*1 (HMGXB3)*
IgA nephropathy		*1 (ITGAX)*
IgG glycosylation		*4 (CHD9, CRBN, IKZF1, SUV420H1)*
Immune response to smallpox vaccine		*2 (BCR, CRBN)*
Inflammatory bowel disease		*4 (IKZF1, IKZF3, SMAD3, SYK)*
Multiple sclerosis		*1 (SYK)*
Omenn syndrome	DCLRE1C * (MIM 603554, AR)	
Platelet count		*1 (BRD3)*
Primary biliary cirrhosis	*NOTCH1, EMC10*	
Psoriasis		*1 (SNAI1)*
Psoriasis and Crohn’s disease combined	*NOTCH1*	
Response to tocilizumab in rheumatoid arthritis		*1 (CCNG2)*
Rheumatoid arthritis	*DCLRE1C*	*4 (ARAP1, ATM, C11orf54, PFKL)*
Selective immunoglobulin A deficiency		*1 (SIRPB1)*
Self-reported allergy		*4 (IKZF1, IKZF3, RANGAP1, SMAD3)*
Severe combined immunodeficiency with sensitivity to ionizing radiation	DCLRE1C * (MIM 602450, AR)	
**Systemic lupus erythematosus**		***4 (IKZF1, IKZF3, ITGAX, SNAI1)***
**Systemic lupus erythematosus and systemic sclerosis**		***1 (IKZF3)***
Type 1 diabetes	*DCLRE1C*	*2 (PRKD2, SIRPB1)*
Ulcerative colitis	*NOTCH1*	*1 (IKZF3)*
Wegener’s granulomatosis		*1 (RING1)*

Genes associated with SLE (in bold), autoimmune diseases, and other immune-related phenotypes according to published GWAS studies. Genes related to diseases according to OMIM Morbid are indicated with a star (*). MIM phenotype numbers are specified between brackets. AD = autosomal dominant, AR = autosomal recessive, S = somatic. See details and references in Table S6.

#### Functional Enrichment

**Figure 6:**
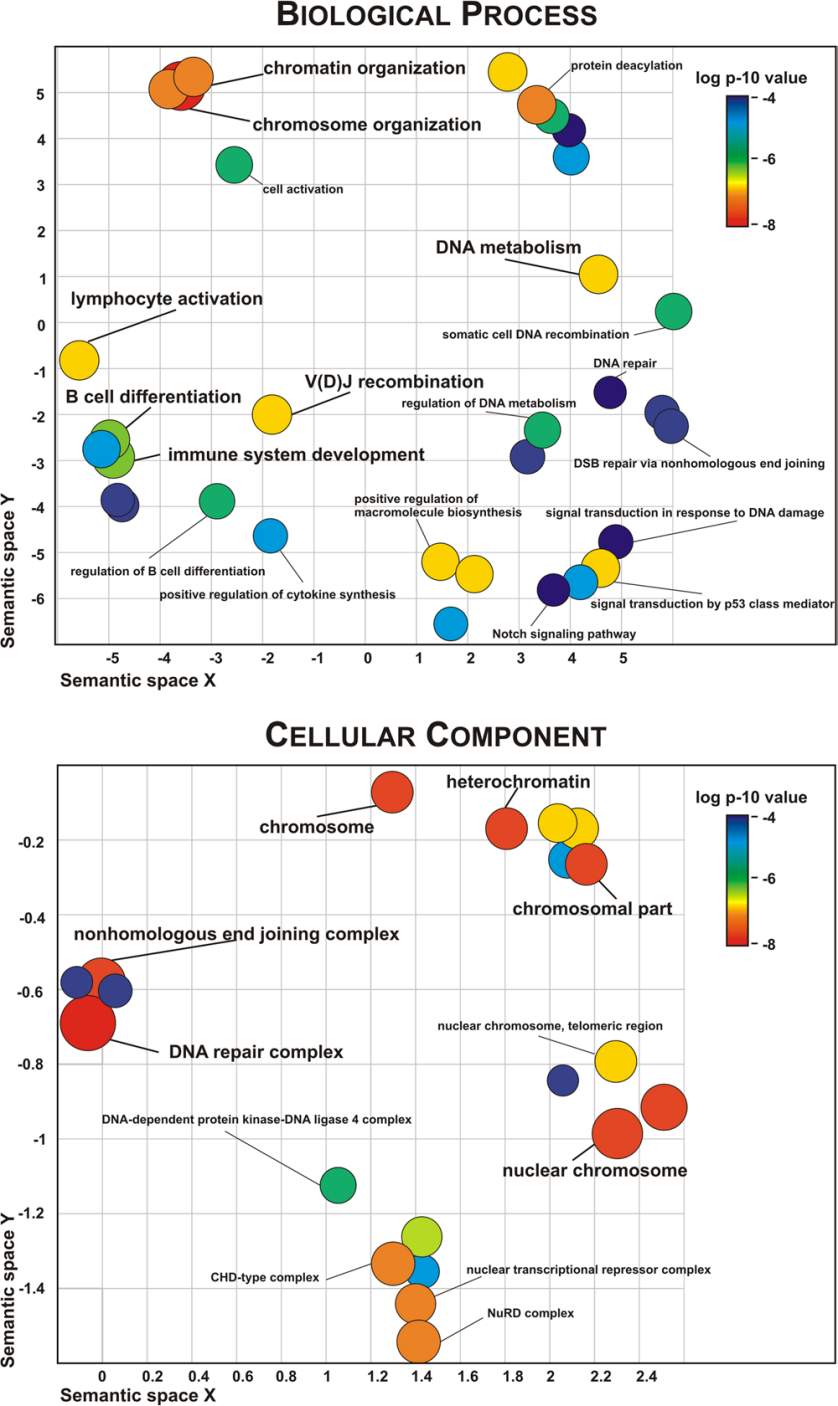
GO categories enrichment for genes with SLE-associated nsSNVs in multicase families and their interaction partners according to TOPPGENE. The overrepresented GO terms were summarized by REVIGO to representative subsets of terms using a simple clustering algorithm that relies on semantic similarity measures. The scatterplots are shown for Biological Processes and Cellular Component, the circles correspond to the cluster representatives (i.e. terms remaining after the redundancy reduction) in a two dimensional space derived by applying multidimensional scaling to a matrix of the GO terms’ semantic similarities with an allowed similarity threshold = 0.9. The colour code and the size of circles reflect p-value of the overrepresented term (Table S7).

To further understand the functional relevance of the identified genes, we performed an enrichment analysis for categories using two independent tools: GeneTrail2^41^ and TOPPGENE^42^. We interrogated the list of genes with nsSNVs identified in the multicase families first, and then we included the stable interaction partners identified in gene networks. We found that functional categories related to DNA metabolism and repair were significantly enriched in the exome gene list (*XRCC6BP1*, *DCLRE1C*, p_(FDR-corrected)_ = 1.08 × 10^−4^, according to TOPPGENE), with the most significant results for V(D)J recombination (*DCLRE1C*, p_(FDR-corrected)_ = 5.8 × 10^−6^). Several immunity-relevant biological processes were also among the top enriched functional categories, including immune system development (*NOTCH1*, *DCLRE1C*, *CLC*, p_(FDR-corrected)_ = 1.7 × 10^−6^), and lymphocyte activation (*DCLRE1C*, *CLC*, p_(FDR-corrected)_ = 6.5 × 10^−6^) (Fig. 6 and Table S7). Of note, when including mouse phenotype data in the analysis, we observed highly significant enrichment of a phenotype of absent immature B cells (*DCLRE1C*, p_(FDR-corrected)_ = 5.3 × 10^−6^). GO cellular component ontology analysis also revealed overrepresented functional groups: genes associated with DNA repair complex and nuclear chromosome. Figure 6 shows a semantic grouping of the most significant GO categories overrepresented in the list of our SLE candidate genes and their interaction partners and summarizes graphically the top enriched categories (shown also in Table S7).

#### Variants Putatively Linked to Gene Regulation

We observed that some of the exome variants were located in gene regulatory regions and thereby could play a role in the regulation of their respective genes. The *EMC10* gene is located head-to head and shares a short bidirectional promoter with *FAM71E1*. According to RefSeq and GENCODE v.19 annotation, the *FAM71E1* 5′-UTR overlaps with the *EMC10* transcription start site and the first exon. These genes are in a genomic region with very strong LD. As mentioned before, unlike *FAM71E1*, whose expression is restricted to testis, *EMC10* has a broad expression pattern. Using Haploreg^43^ for 1000 Genomes European data, we found that the *FAM71E1* variant (p.L7F) (rs185418641) and its proxies are located in an open DNAse-hypersensitive chromatin site in various blood cell types. *FAM71E1* is not expressed in those cell types, in contrast to *EMC10*.

The *SLC5A9* variant (p.G103R) (rs61746559) corresponds to a immune-relevant enhancer detected in two independent functional genomics projects (FANTOM5, based of CAGE sequencing and Roadmap Epigenomics, based on histone marks). In contrast to the *SLC5A9* gene, this enhancer was active in the blood, mostly in neutrophils, but not in gastrointestinal samples, similar to immune-relevant expression of candidate SLE genes.

We found significant eQTLs markers in *SLC5A9* (9.76E-07, rs850762) and *EMC10* (rs921938, p-value 7.62E-67) genes in BIOSQTL whole blood eQTL browser^44^ (https://molgenis26.target.rug.nl/downloads/biosqtlbrowser/). However, the most significant eQTLs are not in LD with the SLE exome variants. We did not find eQTLs neither for *EMC10* nor *SLC5A9* in GTEX data (As to June 29^th^, 2017).

## Discussion

The pursuit of the genetic variants that explain why some individuals and their relatives have an increased risk to develop SLE is a challenging task. In our study, we focused on the identification of rare coding variants with a likely gene-disrupting effect segregating with SLE in two large pedigrees from Iceland for which we have clinical and linkage analysis data. The structure of these families might suggest a Mendelian (monogenic) pattern of inheritance, however, we found no single gene-disrupting variant segregating with the disease status in all family members. Instead, we observed a small set of rare and low frequency (MAF < 1%) coding non-synonymous variants segregating with the disease status in each family.

We thus looked to interrogate whether there was supporting genetic evidence for any of the family genes identified at the population level. We did so by using highly stringent rare-variant association analysis with data from a recent GWAS, a strategy previously used by us that provided support for rare variants in the *RNASEH2* genes^45^. More recently, genetic association of SLE with a common variant in *RNASEH2C* was identified and replicated using this data in a European-Chinese meta-analysis, a variant that represented an eQTL^46^. Initial analysis based on both aggregated association and enrichment analysis of rare and low frequency variants in the identified loci supported the implication of the *FAM71E1/EMC10* locus and possibly *DCLRE1C*. Further *in silico* analysis suggests a role of the variant identified by exome sequencing on the regulation of *EMC10* expression. *EMC10* (ER membrane complex subunit 10) codes for a protein involved in endoplasmic reticulum (ER)-associated degradation and lipid transport, but any role in SLE is completely unknown. The association analysis performed here might have been too stringent, but it allowed us to conclude with high confidence that the enrichment of rare variants at the *FAM71E1/EMC10* locus is robust and not due to the effect of population stratification or LD. Regarding the other genes not associated in the GWAS data, they still might play a role in these families but not at the European population level, taking also into account possible differences between the populations studied. However, we must keep in mind that to date there are no standard methods to detect association of rare variants in complex diseases and this is a field under development with still with many challenges to address^47,48^.

Bioinformatics analysis indicated that a part of the identified variants may be predicted as having a deleterious effect, and for some variants methods did not agree. For example, the variant in *DCLRE1C* (DNA cross-link repair 1C) coding for the nuclear protein Artemis, was predicted as probably benign by standard algorithms but highly deleterious using a one-to-one orthologue specific approach^33^. Further analysis showed though that the detected His- > Asn change is located in a well conserved loop nearby the catalytic center and predicted DNA binding sites (Fig. S7). In addition, analysis of two variants disregarded later due to their high allelic frequency (MAF > 1%) nevertheless indicated them as worth further study: one in the neutrophil-specific enhancer in the intronic area of *SLC5A9* gene and the other in the natural killer-specific *KIR2DS4* gene, one of the KIR family of genes involved in the inhibition and activation of NK cell function that interact with class I MHC molecules^49^. This highlight some of the limitations of this study. First, in the absence of functional evidence, results from variant prediction algorithms can only be taken as suggestive evidence. Second, here we did not evaluate the role of common variants nor have we analysed other types of genetic variation such as structural or copy number variants.

We carried out the analysis of gene networks based on gene co-expression and protein-protein interactions and identified a functional overlap between genes, even for those segregating independently in each family. The constructed gene networks revealed among the exome genes, partners previously implicated in autoimmune diseases, including the SLE-associated genes *IKZF1, IKZF3*, and *ITGAX*^25,50,51^ (Tables 3 and S6), further supporting a role for the genes identified in the disease pathogenic pathways. The importance of multiple factors and gene interactions in the genetics of complex traits is well known, and even minor fluctuations of genes expressed in the disease-relevant cells could probably contribute to the disease susceptibility, as discussed in the recent “omnigenic” hypothesis^52^. We demonstrate that even for SLE in multicase families with apparent Mendelian inheritance the underlying mechanism is complex and involves several functionally interacting genes. In contrast to family studies of Mendelian diseases, we cannot easily address issues such as incomplete penetrance nor the possibility of phenocopies, as to do so we would need to take into account all the possible genetic and non-genetic factors contributing to complex landscape of this disease.

Functional enrichment analysis of the identified genes and their interaction partners demonstrated significant overrepresentation of immunity related terms within GO categories such as DNA repair and DNA metabolism including V(D)J recombination, a critical process in the rearrangement of the T cell and B cell receptors, double strand break (DSB) repair, cellular response to DNA damage stimuli, and chromosome organization. Also, we detected a significant enrichment of genes involved in the NOTCH1 pathway (Fig. 6 and Table S7). The DSB repair and V(D)J recombination categories were represented by *DCLRE1C* and *XRCC6BP1* (XRCC6 binding protein 1) genes and their interacting partners. Of note, *DCLRE1C* recessive mutations cause Omenn syndrome (MIM 603554) (OS), a severe combined immunodeficiency (SCID) associated with increased cellular radio sensitivity due to a defect in V(D)J recombination that leads to early arrest of both B- and T-cell maturation^53^. OS displays autoimmune-like manifestations of the skin and gastrointestinal tract. SNPs in *DCLRE1C* have shown suggestive evidence of association with RA and T1D^54,55^. A recent functional study demonstrated that in Artemis-deficient cells type I and type III IFN signatures are elevated due to the chronic accumulation of DNA^56^.

In addition, a widely expressed heterochromatin gene *MPHOSPH8* (M-phase protein 8), whose protein binds H3K9me and promotes DNA methylation^57^, was connected to *DCLRE1C* in all our networks, both directly and via partners. Another gene participating in chromatin regulation and highly connected to other genes in our networks was *CHD3* (Chromodomain Helicase DNA Binding Protein 3). Autoantibodies against this protein are found in a subset of patients with dermatomyositis, also an autoimmune disease^58,59^. Similarly, and possibly related through *XRCC6BP1*, the *XRCC6* gene codes for the Ku70 helicase and V(D)J recombination repair protein, a well established lupus autoantigen^60^.

Our results contribute to the growing evidence linking SLE to DNA damage and repair mechanisms (reviewed in^61^). Increased DNA damage and radiosensitivity have been consistently reported in cells from SLE patients^61,62^. Abnormalities in V(D)J recombination in individuals with combined immunodeficiency carrying hypomorphic *RAG1* pathogenic variants show manifestations of autoimmunity^63^. Importantly, abnormalities in enzymes involved in DNA metabolism have been implicated in the type I IFN response and the development of autoimmunity^64^. For example, several other genes involved in DNA repair pathways have been previously found associated with SLE: *TREX1* (3′ repair exonuclease 1)^65,66^ and X-Ray Repair Complementing Defective Repair genes including *XRCC1, XRCC3*, and *XRCC4*^67,68^.

Finally, we detected a significant enrichment of genes involved in the NOTCH1 pathway. We did not detect association of rare variants in the *NOTCH1* gene itself but previous GWAS have implicated polymorphisms in this gene with several autoinflammatory diseases (Tables 3 and S5)^69–71^. It is worth to note also that somatic recurrent mutations in *NOTCH1* and *NUP214* are found in patients with hematologic malignancies for which SLE patients have an increased risk compared to the general population^72–74^.

In summary, we identified novel SLE susceptibility genes using exome sequencing of distantly related patients from extended pedigrees from Iceland. Taking into consideration the genetic co-segregation of variants, similar gene expression patterns, results of nsSNV protein effect prediction and gene networks modeling, we propose joint multigene mechanisms of SLE predisposition in these families. These genes highlight a role for DNA metabolism and repair in SLE pathogenesis.

## Subjects and Methods

### Patients and Families

We sequenced the exome of the most distantly related patients from two previously described SLE multi-case families from Iceland for which genetic linkage data was available^31^ (Fig. S1). Both families have been extensively studied and have multiple cases of SLE as well as a high frequency of other autoimmune diseases^75^. All patients fulfilled the 1997 ACR classification for SLE^76^. The National Bioethics Committee (NBC) of Iceland approved the study (Approval: 02022-V4-31) and all participants gave informed consent. All experiments were performed in accordance with relevant guidelines and regulations. The details of each exome-sequenced individual and clinical data are provided in Table S1. Two SLE patients were selected from “Family 6” and three from “Family 8” (n = 5). The linkage evidence involved regions with LOD scores of 1.5–4.5. Family 8 contributed the most to the genetic linkage signals observed in the Icelandic linkage study^31^ including the HLA. Family 6 did not show linkage to the HLA region^31^, thus suggesting that the genetic contribution in both families was different.

### Exome Sequencing

Three micrograms of genomic DNA purified from blood of the five selected patients were enriched for coding regions using the Agilent SureSelect® Human All Exome Target Enrichment System (38MB and 51MB kit, protocol v1.7). Deep sequencing was performed at Uppsala University on an ABI SOLiD™ 5500xl system (Life Technologies). Colour space read correction and alignment to the Human reference sequence library (hg18) were performed at the Centro Pfizer-Universidad de Granada-Junta de Andalucía de Genómica e Investigación Oncológica (GENYO) with SOLiD™ Bioscope Software (v.2.1, Life Technologies), obtaining a mean read depth of 33.3X across targeted coding regions (see Supplemental data and Table S2).

### Variant Calling, Annotation and Filtering

We selected only reliably mapped reads with a mapping quality (MAPQ) value over 20. PCR duplicates were removed with Picard (v1.35). Single nucleotide variants (SNVs) and indels were called by using SAMTOOLS (v0.1.10) and then exported to pileup files. Indels were called but not included in the present study because the difficulties this posed with the very short reads obtained. Information about the population frequency of Indels in public databases was also very limited at the time we selected variants for validation. SNVs in pileup file format were annotated and filtered at the Center for Human Genome Variation, Duke University, by using the Sequence Variant Analyzer (SVA) software developed by Dongliang Ge^77^. Whole genome sequence (WGS) data from 642 Caucasian unrelated individuals without any immune-related phenotype served as internal control genomes. Annotated variants in cases were annotated and filtered using the same quality control criteria and methods as the controls. Only SNVs supported by a minimum of 6 reads were included. Variants situated within UCSC Genome browser repeat masker regions were excluded. The alternative allele was compared to the chimpanzee reference allele to ensure none of the alternative alleles represent the expected ancestral allele. Tables S8 and S9 contain the summary statistics for all the shared variants filtered by SVA. The average read depth of the filtered variants in the patients was 51.44X (range 12X–180.79X).

We then selected variants by function and MAF as follows: Only protein-coding variants either introducing or removing a stop codon (*stop gained* and *stop lost*, respectively), altering a splice acceptor or splice_donor_site (*essential splice site*), or introducing an amino acid change (*non-synonymous coding*) were included. SVA uses standard Sequence Ontology (SO) definitions (http://www.ensembl.org/info/genome/variation/predicted_data.html). We considered SNVs shared between the patients of each family, with a MAF ≤ 0.5% in the control genomes. We also considered SNVs that were absent in the controls (MAF = 0), that is, those carried only by the patients (*case-only variants*), as well as SNVs carried in homozygous state exclusively by the patients and present in the control population only in heterozygous state with a MAF ≤ 5% (*case-only-homozygous variants*). We annotated variants present within the Icelandic SLE linkage regions^31^ (+/−20MB) by using BEDTools. Finally, after variant validation, we also compared the allele frequencies obtained with the allele frequencies observed by the NHLBI GO Exome Sequencing Project (ESP6500) (European American population), The Exome Aggregation Consortium (European-non Finnish population) and the 1000 genomes project (Phase 3 European population) by using ANNOVAR v2012 Oct23^78^. This strict filtering strategy favours a reduction in false positives and has been widely and successfully used for the identification of disease variants in Mendelian disorders^79,80^. For further genetic analysis, the genes where the selected variants are located were referred to as candidate genes.

### Segregation Analysis

The identified SNVs were genotyped on a MassARRAY System (SEQUENOM) in: affected as well as healthy members from the multi-case families for whom DNA was available (Fig. 1**)** in 83 matched Nordic controls (n = 36 Icelandic and n = 46 Swedish controls) to determine if any of the variants were polymorphisms (MAF > 5%) specific of the North European population, and in the exome-sequenced patients as genotype controls (n = 5). We selected only individuals with a call rate per sample ≥80%. In total, six patients with SLE, one with rheumatoid arthritis (RA), one with multiple sclerosis (MS), and 1 healthy relative were included from family 6 (n = 9). Five patients with SLE, 2 individuals fulfilling 3 of 4 SLE criteria, and 4 healthy relatives were included from family 8 (n = 11). We selected only variants with a genotyping call rate ≥90% in all genotyped individuals and for which the alternative allele identified by WES was validated in the exome-sequenced patients. As we needed complete genotypes for segregation analysis, missing genotypes for the filtered variants were completed with Sanger sequencing.

### Genome-Wide Association Analysis

#### SNP Data

We used previously genotyped GWAS data from 5,478 individuals of European ancestry including 4,254 SLE patients and 1,224 controls genotyped as described in^25^ using the Illumina© HumanOmni1_Quad_v1-0_B chip and 3,125 out-of-study controls of European origin obtained from three studies available through dbGaP with informed consent, namely the DCEG Dataset (phs000396.v1.p1; 1175 individuals), the GENIE UK-ROI Diabetic Nephropathy GWAS (phs000389.v1.p1; 903 individuals) and the High Density SNP Association Analysis of Melanoma (phs000187.v1.p1; 1047 individuals). The final data set used for aggregate/association analysis consisted of 4,212 cases and 4,065 controls (see Supplemental data).

#### Imputation

For each disease candidate gene, a region of interest was extended in 500,000 additional base pairs upstream and downstream, respectively, as it is known that large buffers may improve accuracy for low-frequency variants during imputation^81^. Markers within each extended region were extracted from the GWAS data for imputation with IMPUTE2^82^ using the 1000 Genomes Project as reference panel. Specifically, we used 1000 Genomes Phase 3 (b37) as these haplotypes have lower genotype discordance and improved imputation performance into downstream GWAS samples, especially at low frequency variants^83^. Prior to imputation, each GWAS gene extended region was phased with SHAPEIT^81^ using the EUR subpopulations as reference. A restrictive QC-filter was applied on the imputed genotypes (SNP genotyping rate ≥99%, sample genotyping rate ≥95%) without restriction of allele frequencies, in order to include both rare and low frequency variants. To ensure a highly reliable imputation, a conservative IMPUTE *info_value* threshold of ≥0.7 and a concordance value threshold of ≥95% for each marker were applied. We have further addressed potential bias introduced by imputation by using different association methods and keeping a very stringent significance threshold in our analyses.

#### Gene case-control association analysis of rare variation

Since a minor allele frequency (MAF) of 1% or more is the conventional definition of polymorphism^84^, thus we considered a MAF < 1% as ‘rare variation’. We tested whether any of the genes identified by exome sequencing had statistical evidence of association with SLE in the general European population due to the combined effect of all rare variation within each gene (MAF < 1%). For this, and because there was no availability of DNA from large enough sets of patients to be sequenced, we took an alternative approach. We used a large and independent imputed genome-wide association scan from a set of 4,254 SLE patients and 4,349 controls with European ancestry^25^. Each gene was analyzed using two procedures: the sequence kernel association (SKAT) test^32^ and an aggregated case-control enrichment test where adjusting a logistic regression model with a ‘transformed’ genetic variable equals to the sum of minor frequency alleles, below the MAF threshold, for the *j* markers in the gene, in each individual.

#### Correcting for stratification in rare variant association analysis

To be as stringent as possible, the 10 first principal components (PC) accounting for all the significant variance due to population stratification (Figs S2 and S3) and genomic control (GC) were used to correct for stratification in both procedures. The genomic inflation factor (λ_GC_) was equal to 1.11 and 1.24 for ‘enrichment case-control’ and SKAT 10 PC-corrected tests respectively (Fig. S8). These λ_GC_ values were used as an additional correction of the resulting inflation (GC-correction = Statistic10PC’c_corrected/λ_GC_). Without 10 PC-correction the λ_GC_ was equal to 1.44 and 2.97 for ‘enrichment case-control’ and for SKAT respectively (Fig. S8). Thus, the 10 PC-correction reduced the inflation by 33% in the enrichment test, and by 174% in the SKAT test.

#### Correcting for multiple testing in gene case-control association analysis of rare variation

Regarding the correction for multiple testing in association of rare variants, a genomic association threshold of 10^−6^ is commonly accepted (equivalent to Bonferroni correction for 19,000 to 20,000 protein encoding genes in the genome). It is also accepted that Bonferroni, although mathematically right, would be very penalizing for biological data, therefore, we opted for techniques based on permutation procedures. Our multi-test correction procedure brings together all the markers of the analysed genes into a single table; for each gene a number of markers equal to that of the analysed gene were randomly extracted from the table and its association test calculated; by repeating the procedure for N times, an empirical corrected P value (*Pmult*) was calculated for the analysed gene. However, in association tests that simultaneously include several markers, co-linearity due to LD between markers could potentially inflate the significance of the P value. Also, elimination of the LD by the random extraction of markers in the permutation procedure could affect the computation of empirical corrected P values. A simple correction to this is to run the association and the multiple test correction tests only with unlinked markers by applying a very stringent LD threshold of r² < 0.1. These corrected P values would depend on the observed P values used as thresholds. We have verified by linear correlation (P corrected multi-testing ~ P observed) that R² was equal to 0.99 for the enrichment tests and 0.97 for the SKAT tests. Thus the LD would not affect the correction, as expected, given the applied r² threshold.

It could be objected that applying such strict filers could mask true association signals, but given the issues related to the association of rare variants^85^, we reasoned that under all these stringent criteria any significant association signal would strongly support a real or ‘true positive’ association effect.

### Functional Bioinformatic Analysis

#### Annotation of SNV Effects on Protein Function

ENSEMBL VEP (Variant Effect Predictor)^34^ was used for the annotation of the potential deleterious effects of the exome variants based on the following algorithms: SIFT, PolyPhen2, FATHMM, LRT, MetaLR, MutationAccessor, MutationTester, and Provean. None of the applied methods was adapted for distinguishing effects in the paralogous proteins. Further, functional prediction of SNVs on protein-coding ENSEMBL transcripts was performed using the SNPDryad method^33^ (http://snps.ccbr.utoronto.ca:8080/SNPdryad/), for which only one-to-one orthologous proteins were used for scoring. An SNPDryad score below 0.5 was assigned as possibly neutral, from 0.5 to 0.7 as possibly deleterious, and more than 0.7 as deleterious. To deal with multiple annotations, gene transcripts were first scanned for isoform-specific expression level in the GTEx Portal^86^ (http://gtexportal.org). Only the main protein-coding isoforms were selected for the annotation of putative gene-damaging effects. The analysis was based on the Human Genome annotation GRC38_p3.

#### Annotation of Nearby Variants Associated in GWAS

All known genetic associations were parsed using GRASP GWAS database^87^ (http://grasp.nhlbi.nih.gov) and NHGRI-EBI GWAS Catalogue^88^ (https://www.ebi.ac.uk/gwas/home). For each identified exome variant, 100 kb and 500 kb-flanking genomic regions were scanned for the presence of published immune-relevant GWAS markers (p-value = <10^−7^). Genes associated with known monogenic disorders were searched using OMIM database^89^ (http://www.omim.org/). The relevance of the phenotype to immunity was parsed based on the Associated Human Phenotype HPO classes using the HPO Browser^90^ (http://human-phenotype-ontology.github.io/tools.html).

#### Tissue-specific Expression and Gene Regulation Analyses

The differential expression of genes including tissue-specific and from eQTL profiling was analysed using aggregated public microarray and RNAseq data available at GTEx^86^ and MuTHER^91^. The TSS activity and gene enhancers were studied based on the FANTOM5 project CAGE data^92,93^, which contains about 900 tissue and cell specimens (http://fantom.gsc.riken.jp/5/). The regulatory enhancer elements and their tissue-specific activity were recovered from Roadmap Epigenomics data^94^ (http://www.roadmapepigenomics.org/). The differential expression data were extracted from the EMBL-EBI Expression Atlas^95^, (https://www.ebi.ac.uk/gxa/about.html) Only relevant differential expression results, involving immunity, inflammation, immune- or inflammatory-stimuli and cell activation, infections, cell exposure to bacteria or bacterial components. The adjusted p-value threshold was selected as <0.05 and the absolute value of log2 fold-change as > = 2.5.

#### Gene and Protein Network Analyses

Gene-gene pairwise networks were constructed using two main data sources: gene co-expression data and protein-protein interactions. Gene pairs detected in two or more of the co-expression data sets were selected and included in the network analysis. Protein pairs detected in two or more sources were also included in the network analysis (See Supplemental data). Combined co-expression and protein-protein pairwise interactions were searched for direct pairs between query genes or indirect, through gene/protein partner (only one node between query genes allowed). Family-specific sub-networks were also constructed. Tissue-specific gene relations were extracted from the GIANT database^37^ (http://giant.princeton.edu/; See Supplemental data).

#### Gene Functional Enrichment Analysis

A functional enrichment analysis was performed using TOPPGENE Suite^42^ (https://toppgene.cchmc.org) and GeneTrail2^41^ (http://genetrail2.bioinf.uni-sb.de/) Genomics tool. The significantly enriched GO categories were visualized using REVIGO^96^ (http://revigo.irb.hr/).

### Data availability

In adherence with the confidentiality requirements by The National Bioethics Committee (NBC) of Iceland, individual sequences or genotype data cannot be publicly shared. All summary data generated or analyzed during this study are included in the article and its Supplementary Information files.

## Electronic supplementary material

Supporting Information

Table S3

Table S4

Table S5

Table S6

Table S7

Table S8

Table S9
