# Supplementary material for: Whole Exome Sequencing of Patients from Multicase Families with Systemic Lupus Erythematosus Identifies Multiple Rare Variants
Source: Sci Rep. 2018 Jun 8;8:8775. doi: 10.1038/s41598-018-26274-y (PMC5993790; doi:10.1038/s41598-018-26274-y)
Supplement: Supplementary file 1 — Supporting Information [file 41598_2018_26274_MOESM1_ESM.doc]

**Whole Exome Sequencing of Patients from Multicase Families with Systemic Lupus Erythematosus Identifies Multiple Rare Variants**

# Supporting Information

Angélica M. Delgado-Vega,^1,π^ Manuel Martínez-Bueno,^2,π^ Nina Y. Oparina,^3,4,π^ David López Herráez,^5^ Helga Kristjansdottir,^6^ Kristján Steinsson,^6^ Sergey V. Kozyrev,^4,&^ and Marta E. Alarcón-Riquelme^2,3,&,*^

^1^Department of Immunology, Genetics and Pathology, Uppsala University, The Rudbeck Laboratory, UPPSALA, Sweden, ^2^Pfizer/University of Granada/Andalusian Government Centre for Genomics and Oncological Research (GENYO), GRANADA, Spain, ^3^Institute for Environmental Medicine, Karolinska Institutet, Solna, Sweden, ^4^Science for Life Laboratory, Department of Medical Biochemistry and Microbiology, Uppsala University, UPPSALA, Sweden, ^5^Department Effect-Directed Analysis, Helmholtz Centre for Environmental Research - UFZ, LEIPZIG, Germany, ^6^Unit of Rheumatology, Landspitalinn, REYKJAVIK, Iceland.

*Corresponding author

E-mail: [marta.alarcon@ki.se](mailto:marta.alarcon@ki.se)

^π^These authors contributed equally to this work

^&^These authors also contributed equally to this work

# Supplemental Subjects and Methods

## Genome-Wide association analysis

**SNP data.** We used previously genotyped GWAS data from 5,478 individuals of European ancestry including 4,254 SLE patients and 1,224 controls genotyped as described in [^1^](#_ENREF_1) using the Illumina HumanOmni1_Quad_v1-0_B chip and out-of-study controls of European origin obtained from three studies available through dbGaP with the appropriate approvals: (1) DCEG Imputation Reference Dataset (phs000396.v1.p1), 1175 individuals; (2) GENIE UK-ROI Diabetic Nephropathy GWAS (phs000389.v1.p1), 903 controls; and (3) High Density SNP Association Analysis of Melanoma (phs000187.v1.p1), 1047 controls. So the initial dataset consisted of 4254 affected and 4349 controls. In order to obtain a quality-controlled working dataset satisfying current state-of-the-art criteria for association studies, data filtering was conducted using PLINK v1.07 1 (7) applying the following criteria: minimum total call rate per sample of 90%, minimum call rate per marker of 98%, minor allele frequency (MAF) threshold of 0.01%, Hardy-Weinberg Equilibrium (HWE) p-value for cases and controls at a minimum of 0.0001, and in addition at 0.01 only for controls, and finally a cut-off p-value of 0.00001 for differential missingness in no-call genotypes between cases and controls. To analyse cryptic relations between pairs of individuals REAP was used (8) applying a kinship coefficient threshold < 0.055. The final data set used for association analysis consisted of 4212 cases and 4065 controls. Principal component analysis was performed with smartpca, EIGENSOFT 4.0beta package 2 (9).

## Gene and protein networks analyses

Gene-gene pairwise networks were constructed using two main data sources: gene co-expression data and protein-protein interactions. Gene pairs detected in two or more of the co-expression data sets were selected and included in the network analysis. The following criteria for each set were used: (1) biological process-based GO weighting; up to 100 potential related genes; 287 published co-expression only data for human genes in GeneMania compilation (GMX) (http://www.genemania.org) [^2^](#_ENREF_2); (2) Pearson correlation >0.3; mutual rank<500 for GeneFriends RNAseq (GFR) (http://www.genefriends.org/RNAseq/); and various sets at GeneVestigator: (3) all samples (only if >5 samples in category), gene expression perturbations, top 200 genes with Pearson threshold 0.55, (GV1); (4) immune & blood cells samples (only if >5 samples in category), top 200 genes with Pearson threshold 0.8, [anat/neopl/cell line: anatomy-(tissues-organ system-haemalymphoid system), (primary cells-hematopoetic and immune system cell) + cell line-lymphoblastoid cell lines], (GV2); (5) relevant perturbations (only in >5 samples in category), top 200 genes with Pearson threshold 0.7, [perturbations: compound-biological-(antibody-antigen-decoy receptor);(LPS) + disease-(immunology and rheumatology);(infectiology) + infection + other-(allergen exposure)], (GV3) and (6) same as GV1, but "per samples" and threshold 0.8 (GV4).

Protein pairs detected in two or more sources were included in the network analysis. The protein-protein interaction data was integrated using BIOGRID Protein interactor v3.4 (http://thebiogrid.org/); iRefWeb non-redundant human PPI database (http://wodaklab.org/iRefWeb/), (IR); human binary interactome project data, (http://interactome.dfci.harvard.edu/H_sapiens/index.php) (HID) and direct physical interactions only in GeneMania compilation of 190 published PPI experiments (GMP).

Combined co-expression and protein-protein pairwise interactions were searched for direct pairs between query genes or indirect, through gene/protein partner (only one node between query genes allowed). Family-specific sub-networks were also constructed. Tissue-specific gene relations were extracted from the GIANT database [^3^](#_ENREF_3). The following tissues were analysed for tissue specific relations: GI01 (all tissues); GI02 (B lymphocyte); GI03 (blood); GI04 (neutrophil); GI05 (monocyte); GI06 (lymph node); GI07 (T lymphocyte). The confidence threshold 0.3 for the minimum relationship was applied for including in the network, and similar pairwise networks were constructed for query genes, as mentioned above.

# Supplemental Figures

*
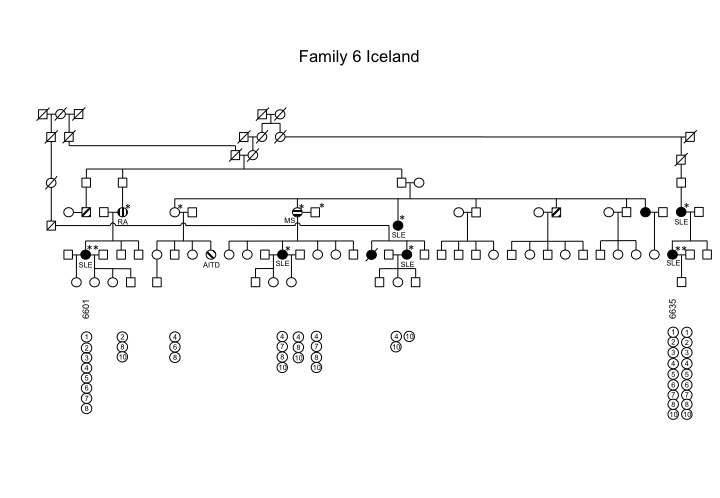

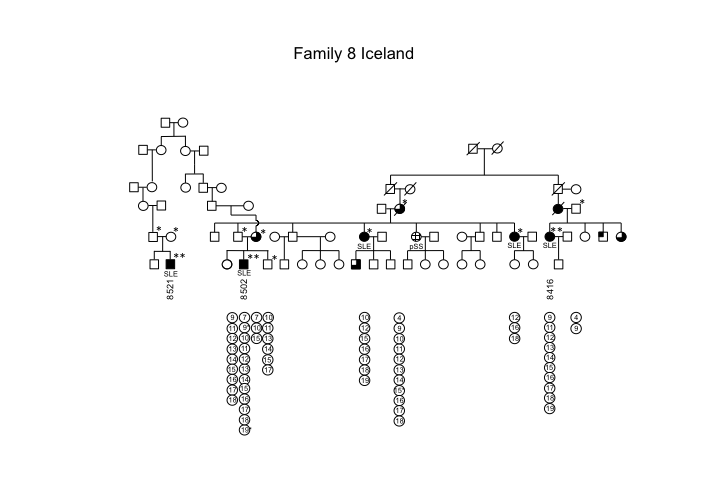
*

**Figure S1. Pedigree of the Icelandic multicase families studied.** Exome-sequenced patients are indicated with two stars (**). Individuals who were genotyped for the identified variants are marked with one star (*). The numbers in circles correspond to the gene variants carried by each individual according to **Table 1**. SLE patients are shown as black-filled circles. Partially filled circles show patients with less than 4 clinical classification criteria. Patients with other autoimmune diseases (family 6, top) are labelled accordingly. RA: rheumatoid arthritis, MS: multiple sclerosis, AITD: autoimmune thyroid disease; pSS: primary Sjögren’s syndrome


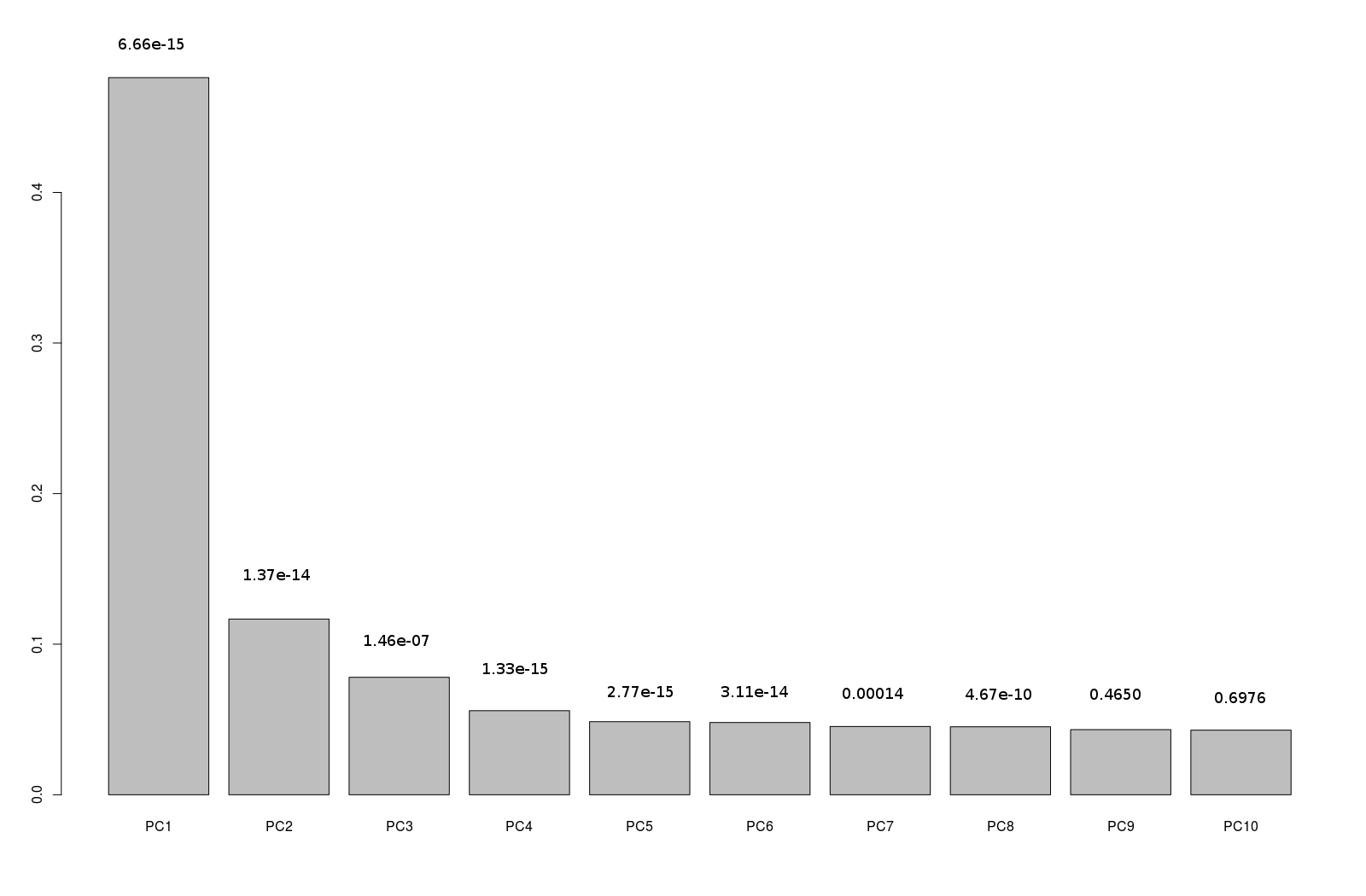


**Figure S2. Proportion of variance explained by each of the first 10 principal components.** The graph shows that only the first 8 PCs (out of 100) were significantly associated with the phenotype (case-control association P values of each PC is over its bar). In other words, they explained all the significant phenotypic variance due to population stratification.


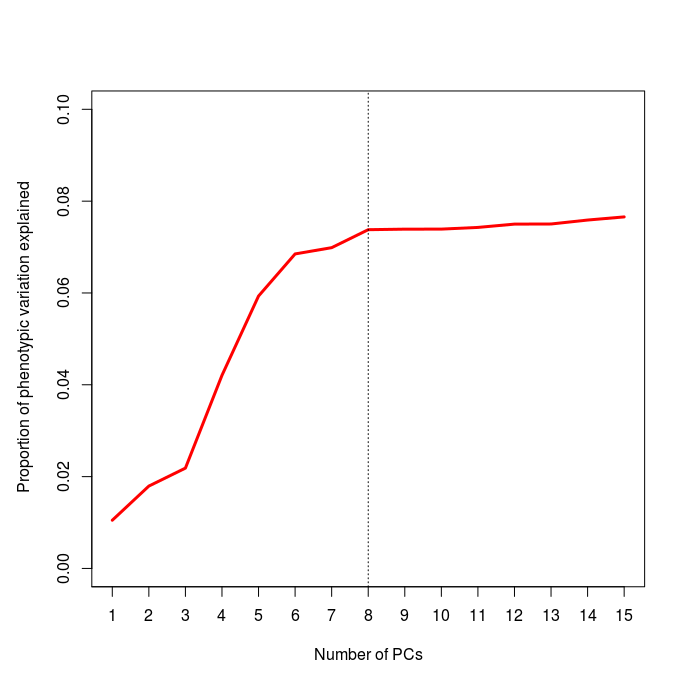


**Figure S3.** **Cumulative proportion of variance explained by the first principal components.** The graph shows that the statistically significant phenotypic variation (7.5%) would be fully explained by the first 8 PC's. Discrimination slope of the adjusted logistic (null) model was used as measure of the proportion of phenotypic variation explained by the PC’s.

*
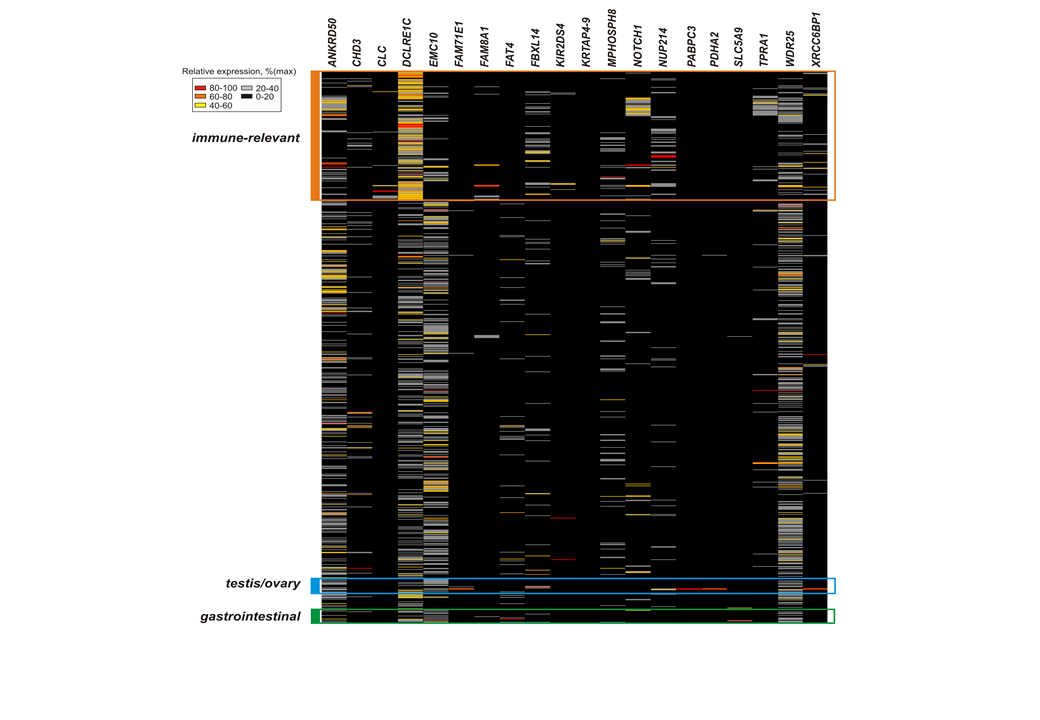
*

**Figure S4.** **The expression levels as defined by FANTOM5 transcription start site (TSS) activity based on CAGE hits.** Groups of immune-relevant, testis/ovary and gastrointestinal tract samples depicted by colored boxes (see **Table S4***).*

**A**

**
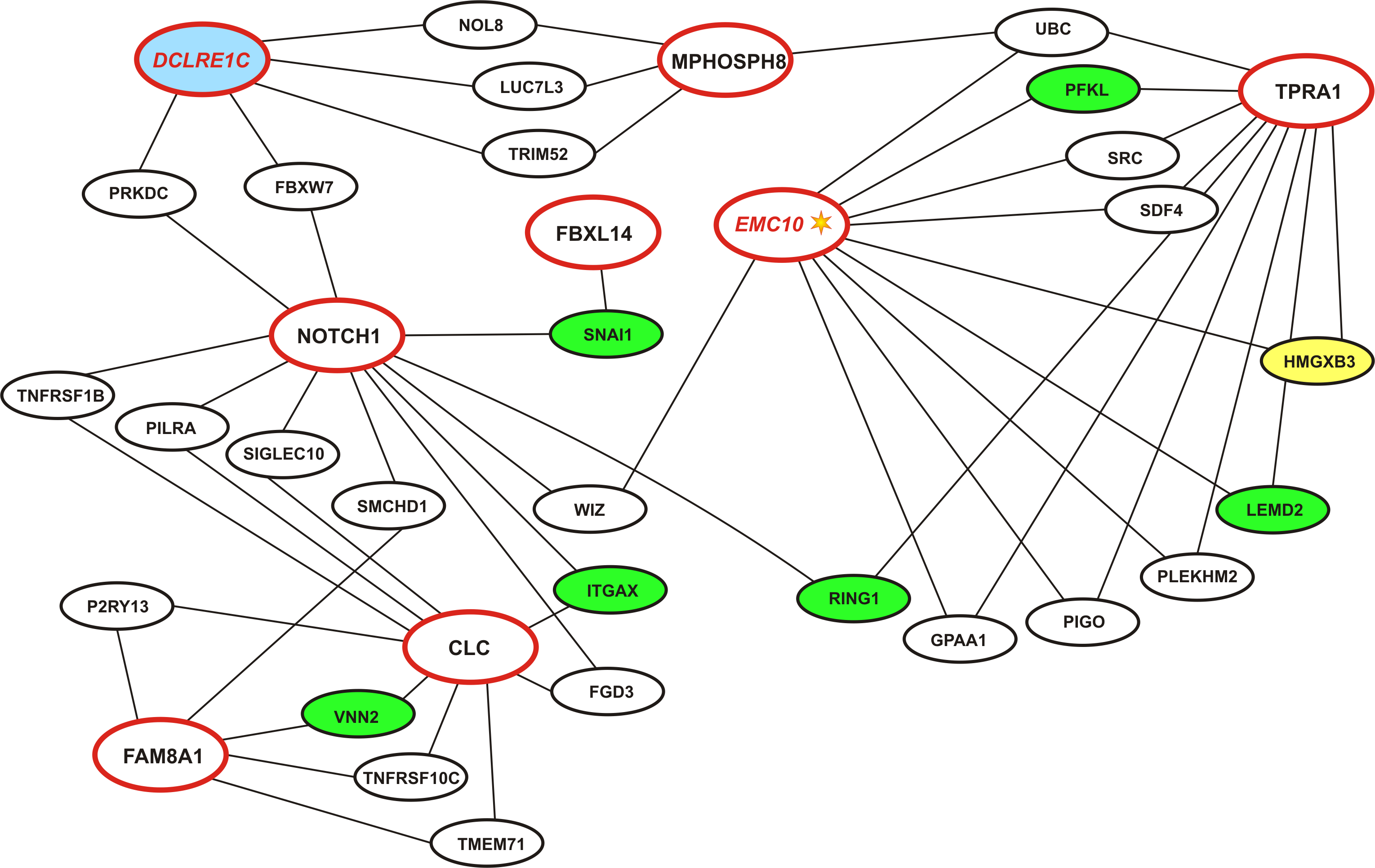
**

**B**

**
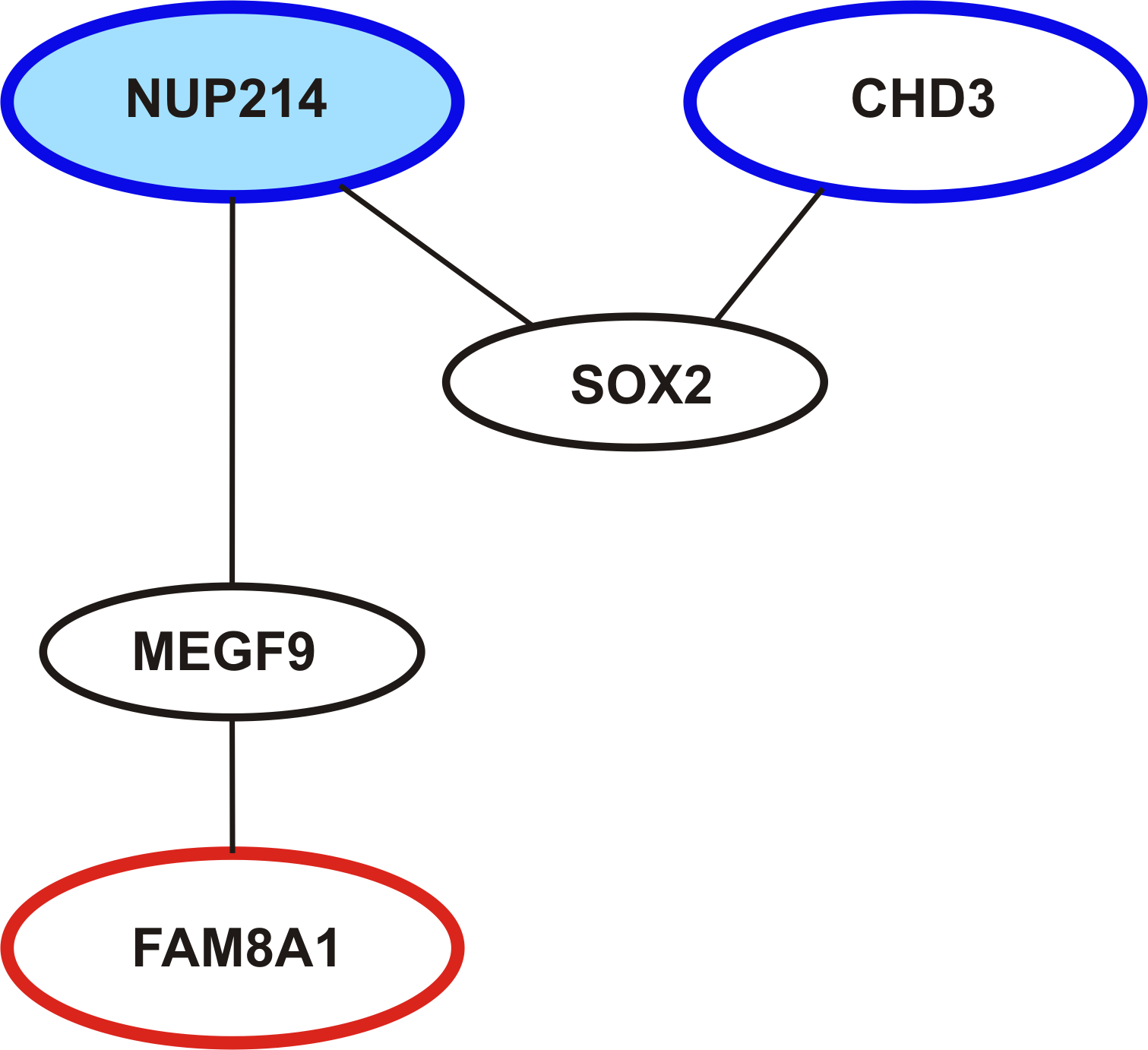
**

**Figure S5. Family-specific gene interaction networks.** Gene co-expression and direct protein-protein interactions are shown as combined networks for each gene and include common partners with other genes. Family 8-specific genes **(A)** are shown in red ovals, family 6 **(B)** - in blue ovals. The red italic gene names correspond to the genes showing significant rare variant enrichment and/or aggregation analysis of sporadic SLE cases. Genes associated after removing linked variants (r^2^<0.1), adjustment by 10 principal components and genomic control, and multiple test correction are highlighted with a star. Blue-filled ovals correspond to the genes with known immune-related Mendelian disorders (OMIM data); green-filled - to the genes with published genome-wide significant associations with autoimmune and autoinflammatory disorders; yellow-filled – with other genome-wide significant immune-related disorders and traits (see **Table S6**).

***
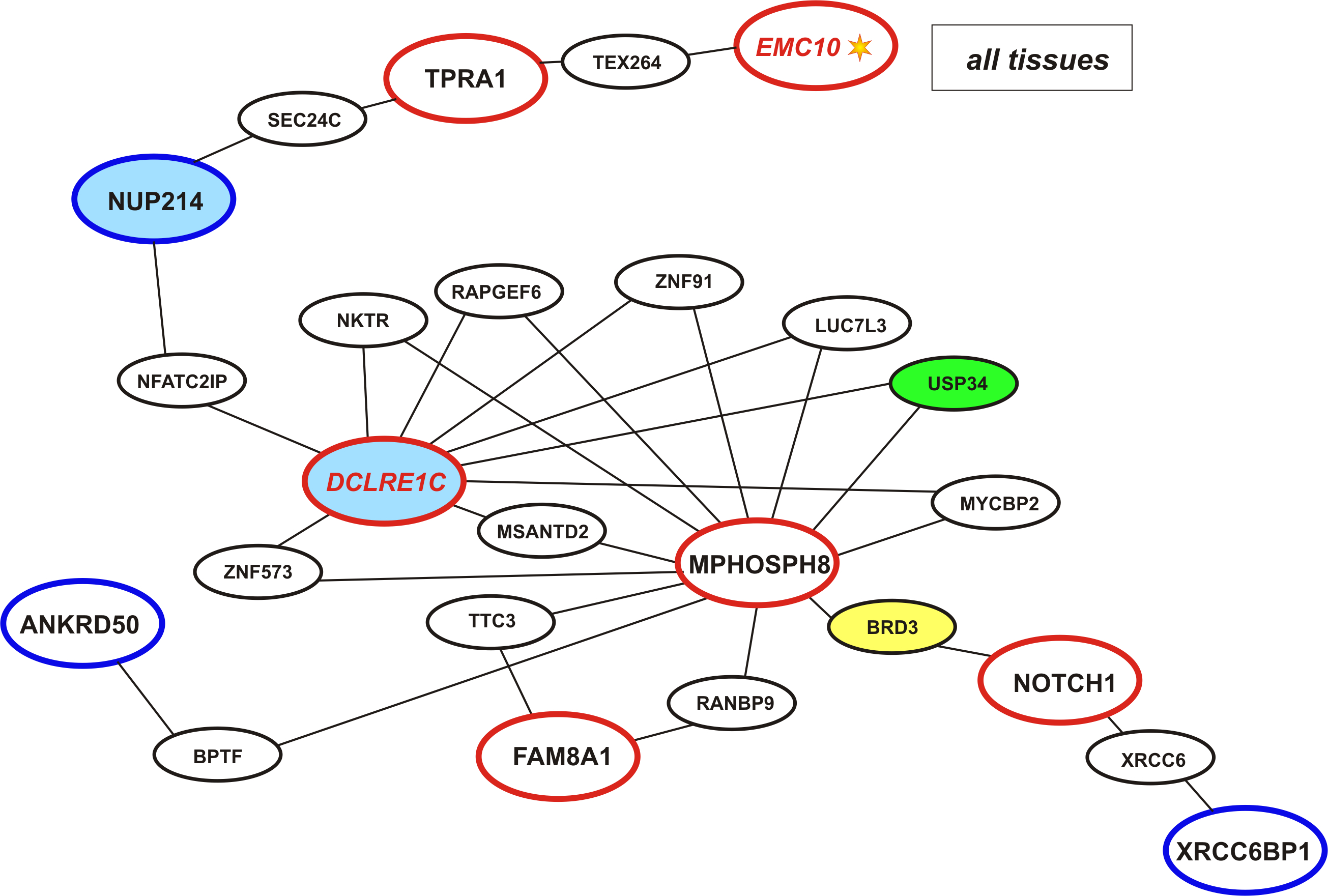
***

**Figure S6.** **GIANT gene connectivity network, all tissues.** Designations are as in Figure 4.

***
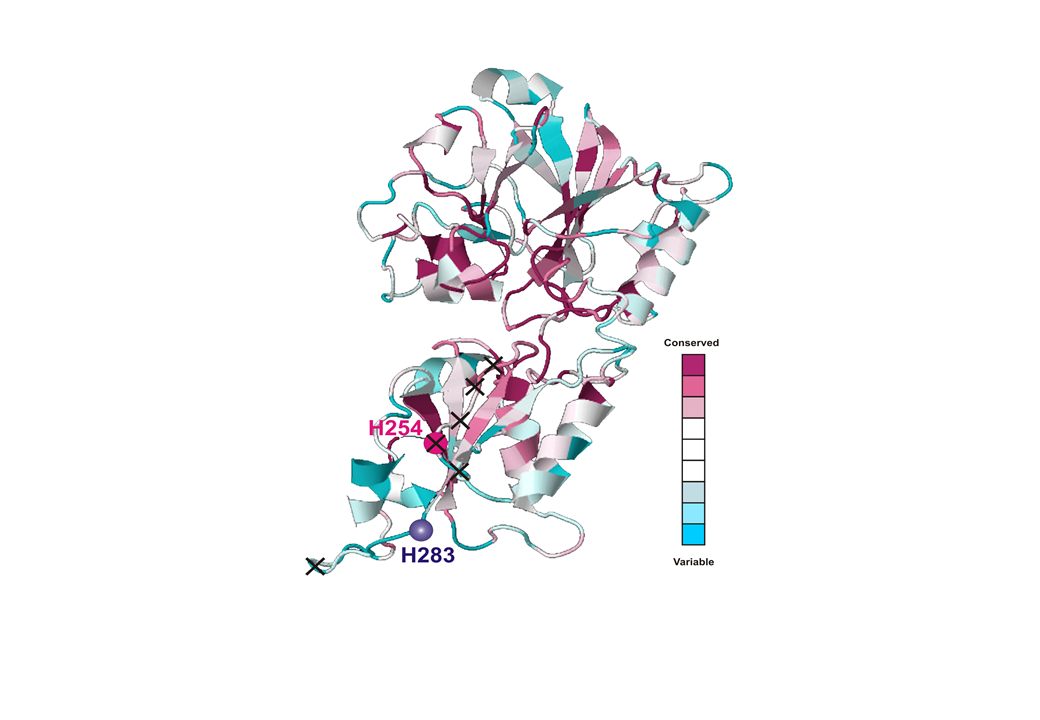
***

***
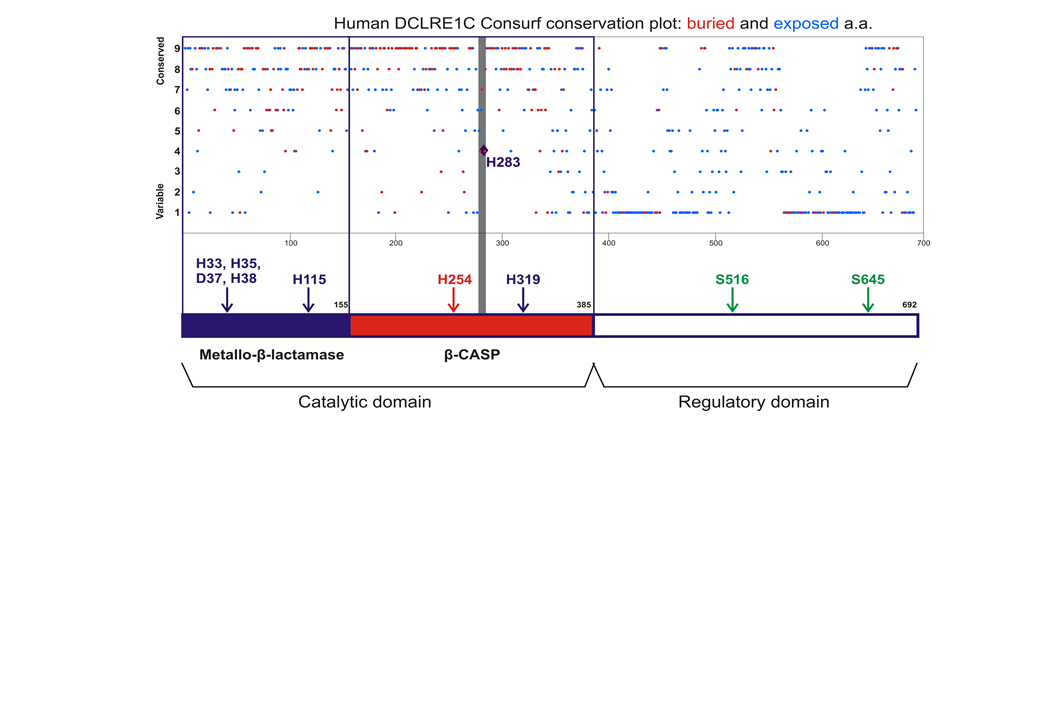
***

**Figure S7. Structural model of the *DCLRE1C* (Artemis) gene and location of the SLE-segregating nsSNV. (A)** The structural representation of the catalytic domain of human DCLRE1C based on the ModBase ([https://modbase.compbio.ucsf.edu](https://modbase.compbio.ucsf.edu/)) molecular model Q5JSS0. Model is considered to be reliable according to database settings. The amino acid chain is colored according to global evolutionary conservation of DCLRE1 family using the ConSurf web server^1^ ([http://consurf2.tau.ac.il/)](http://consurf2.tau.ac.il/) and mapped to the predicted structure. The SLE-associated His283 is shown in violet. The catalytic center residue His254 is shown in red, according to Kurosawa A, et al.^2^ The putative DNA binding residues (shown as black crossed) were predicted using a sequence-based hybrid algorithm SNBRFinder with default parameters at (<http://ibi.hzau.edu.cn/SNBRFinder/about.php)3>. (**B**) Protein functional map. The Consurf scores were estimated as shown above. The nine conservation ranks are shown and colored according to the surface accessibility status of the residues (the buried are red, the exposed are blue). We propose that higher conservation of the exposed residues should be associated with their functional importance, especially in the case of conserved context making the contact interface structurally stable. The known functionally important residues are depicted according to Kurosawa A, et al.^2^ and include the important metal-binding sites (violet arrows), catalytic centre (red) and phosphorylation sites (green).


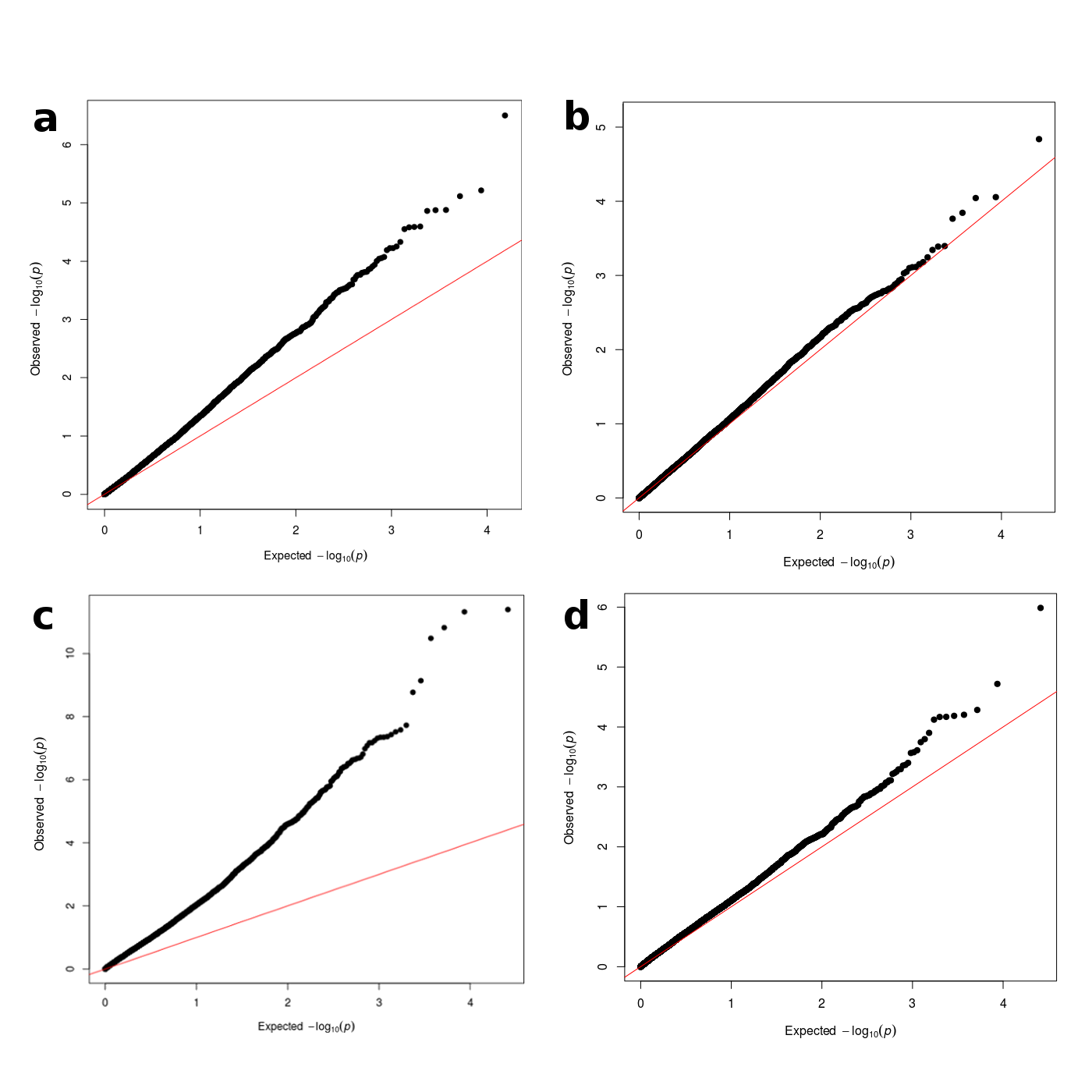


**Figure S8**: (a) QQ-plot of the ‘enrichment case-control’ association analysis without principal components (PC)-correction. Genomic inflation factor (lGC) was equal to 1.44. (b) QQ-plot of the ‘enrichment case-control association analysis’ after 10 PC-correction. Genomic inflation factor (lGC) was reduced from 1.44 to 1.11. (c) QQ-plot of the SKAT analysis without PC-correction. Genomic inflation factor (lGC) was equal to 2.97. (d) QQ-plot of the SKAT analysis after 10 PC-correction. Genomic inflation factor (lGC) was reduced from 2.94 to 1.24.

**S1 Table. Clinical characteristics of exome-sequenced patients.**

| *Severe SLE* | *Renal*  *involvement* | *MultiCase Fam* | *Date of*  *birth* | *Gender* | *Date of diagnosis* | *Date of onset* | *1* | *2* | *3* | *4* | *5* | *6* | *7* | *8* | *9* | *10* |  |  |
| --- | --- | --- | --- | --- | --- | --- | --- | --- | --- | --- | --- | --- | --- | --- | --- | --- | --- | --- |
|  |  |  |  |  |  |  | **Malar** | **Discoid** | **Photosensitivity** | **Oral ulcers** | **Arhtritis** | **Serositis** | **Renal disorders** | **Neurological disorders** | **Hematological disorders** | **Immunological disorders** | **ANA** |  |
| + | + | **6601** | 2/11/56 | F | 1977 | 1971 | 0 | 0 | 0 | 0 | x | x | X | X | x | X | x |  |
| + | + | **6635** | 10/19/56 | F | 1992 | 1991 | x | 0 | x | x | x | X | X | 0 | 0 | X | x |  |
| + | 0 | **8416** | 4/17/50 | F | 1971 | 1971 | x | 0 | x | 0 | x | x | 0 | 0 | x | x | x |  |
| + | + | **8502** | 3/20/72 | M | 1983 | 1980 | 0 | 0 | 0 | 0 | x | 0 | X | X | x | x | x |  |
| X | 0 | **8521** | 1/10/62 | M | 1994 | 1992 | x | X | x | X | x | X | x | X | X | x | x |  |

The IDs of the sequenced individuals are indicated as in the S1 Figure.

**S2 Table. Exome coverage statistics**

DNA from the five patients was enriched for coding regions using the Agilent SureSelect® Human All Exome Target Enrichment System 38MB (8502 and 6601) and 50MB kit (6635, 8416 and 8521)(protocol v1.7). Deep sequencing was performed at Uppsala University on an ABI SOLiD™ 5500xl system (Life Technologies). Colour space read correction and alignment to the Human reference sequence library (hg18) were performed at the Centro Pfizer-Universidad de Granada-Junta de Andalucía de Genómica e Investigación Oncológica (GENYO) with SOLiD™ Bioscope Software (v.2.1, Life Technologies), obtaining a mean read depth of 33.3X across targeted coding regions. Note that we calculated the percentage of reads “on target “using the whole human exome (CCDS) as the target; instead of the regions targeted by the Agilent capture system, which yields lower results but, on the other side, it is a better indicator of the proportion of the human exome that was successfully analyzed.

| *Sample ID* | *SureSelect® Human All Exon kit* | *% of reads mapped to hg19* | *% of mapped reads on target (whole human exome)* | *% of target (whole human exome) with >= 10x coverage* | *Average coverage within target* |
| --- | --- | --- | --- | --- | --- |
| **6601** | 38 Mb | 80.6 | 71.8 | 69.6 | 26.4 |
| **6635** | 51 Mb | 82.9 | 74.7 | 77.6 | 41.8 |
| **8416** | 51 Mb | 85.2 | 76.5 | 74.7 | 35.9 |
| **8502** | 38 Mb | 78.1 | 71.2 | 72.1 | 30.9 |
| **8521** | 51 Mb | 86.4 | 75.8 | 73 | 31.5 |

**Supplementary Tables S3 to S9 are included as separate Excel files.**

**S3 Table. Detected missense variants annotation: protein effect prediction and population frequency.** Human tissue expression data for isoforms shown according to GTEX project.

**S4 Table. Gene expression in human tissues and cells expression for SLE associated genes.** Data shown according to RNAseq-based GTEX project and CAGE-based FANTOM5 project. For the FANTOM5 the expression data is shown for the major transcription start site of each gene.

**S5 Table.** **Gene expression in human tissues and cells expression for SLE associated genes 2.** Data shown according to Gene Expression Atlas (<https://www.ebi.ac.uk/gxa/about.html>) 3116 differential expression experiments in the database (Source: ArrayExpress). Atlas contains data for expression changes using thresholds: absolute value of log2 fold-change > 1 and adjusted p-value < 0.05. We select human data only with the Log2-fold change threshold 2. Only relevant data shown in the table, including: reaction to immune-system or inflammation regulatory stimuli; infections; immune or inflammatory diseases.

**S6 Table. Table SLE-associated genes and their probable interaction partners: known genetic association with human immune-related traits.** Gene presence in the constructed networks is shown. Known genetic data for these genes and their flanking regions with immune-relevant human traits and disorders was shown according to current GWAS databases (GWAS catalog and GRASP database) and OMIM data (inherited only).

**S7 Table. Functional categories enrichment analysis for SLE-associated genes and their probable interaction partners.** Gene lists used for the enrichment analysis were shown. Enrichment data were presented according to GeneTrail2 and ToppGene. Only significant categories were included.

**S8 Table. Summary statistics for all variants shared between the exome-sequenced affected cousins of family 6.** The annotated variant list was generated by the Sequence Variant Analyzer (SVA software). Additional filters on the original list were applied as indicated in detail in Subjects and methods. Briefly, we initially selected variants not common (MAF <1%) in dbSNP and HapMap databases and with potentially deleterious functional categories such as "splice site" (first two nucleotides in exon-intron boundaries), "stop gain", "stop lost", and "non-synonymous coding" that were not present in artifact/repetitive genomic regions. The selected variants were typed in exome-sequenced individuals in a SEQUENOM platform and only variants for which the same genotype was obtained were considered as candidate variants. IND1 is 6601, IND2 is 6635 as in Table S1. Control_MAF refers to the MAF in the internal sequence controls.

**S9 Table. Summary statistics for all variants shared between the exome-sequenced affected cousins of family 8.** The annotated variant list was generated by the Sequence Variant Analyzer (SVA software). Additional filters on the original list were applied as indicated in detail in Subjects and methods. Briefly, we initially selected variants not common (MAF <1%) in dbSNP and HapMap databases and with potentially deleterious functional categories such as "splice site" (first two nucleotides in exon-intron boundaries), "stop gain", "stop lost", and "non-synonymous coding" that were not present in artifact/repetitive genomic regions. The selected variants were typed in exome-sequenced individuals in a SEQUENOM platform and only variants for which the same genotype was obtained were considered as candidate variants. IND1 is 8502, IND2 is 8416 and IND3 is 8521 as in Table S1. Control_MAF refers to the MAF in the internal sequence controls.

***Supplemental references:***

1. Celniker G., et al., ConSurf: Using Evolutionary Data to Raise Testable Hypotheses about Protein Function. ***Isr. J. Chem.*** (**2013)**, 53, 199-206.
2. Kurosawa A., Adachi N. Functions and Regulation of Artemis: A Goddess in the Maintenance of Genome Integrity, ***J Radiat Res.*** **(2010)**, 51(5):503-509
3. Yang X., Wang J., Sun J., Liu R. SNBRFinder: A sequence-based hybrid algorithm for enhanced prediction of nucleic acid-binding residues. ***PLoS ONE.*** **(2015)**, 10(7): e0133260 ).
